# DICE: fast and accurate distance-based reconstruction of single-cell copy number phylogenies

**DOI:** 10.26508/lsa.202402923

**Published:** 2024-12-12

**Authors:** Samson Weiner, Mukul S Bansal

**Affiliations:** 1 School of Computing, University of Connecticut , Storrs, CT, USA; 2 The Institute for Systems Genomics, University of Connecticut , Storrs, CT, USA

## Abstract

Somatic copy number alterations (sCNAs) are valuable phylogenetic markers for inferring evolutionary relationships among tumor cell subpopulations. Advances in single-cell DNA sequencing technologies are making it possible to obtain such sCNAs datasets at ever-larger scales. However, existing methods for reconstructing phylogenies from sCNAs are often too slow for large datasets. We propose two new distance-based methods, *DICE-bar* and *DICE-star*, for reconstructing single-cell tumor phylogenies from sCNA data. Using carefully simulated datasets, we find that DICE-bar matches or exceeds the accuracies of all other methods on noise-free datasets and that DICE-star shows exceptional robustness to noise and outperforms all other methods on noisy datasets. Both methods are also orders of magnitude faster than many existing methods. Our experimental analysis also reveals how noise/error in copy number inference, as expected for real datasets, can drastically impact the accuracies of most methods. We apply DICE-star, the most accurate method on error-prone datasets, to several real single-cell breast and ovarian cancer datasets and find that it rapidly produces phylogenies of equivalent or greater reliability compared with existing methods.

## Abstract (precis)

This work presents two new computational methods for reconstructing tumor cell lineage trees from single-cell copy number data.

## Introduction

Cancer progression is an evolutionary process driven by the accumulation of somatic mutations (Nowell, 1976). Within tumors, there exist divergent subpopulations of cells characterized by distinct sets of somatic mutations, a phenomenon in cancer called intra-tumor heterogeneity (ITH) (Lawson et al, 2018). ITH is a primary obstacle in cancer prognosis, treatment, and prevention, and a better understanding of ITH is thought to be crucial for clinical success (Turajlic et al, 2019). One approach to studying ITH is through elucidating the evolutionary relationships between different cells in the tumor. Typically, the evolutionary history of a tumor is described by a cell lineage tree, where the leaves of the tree represent observed cells in the sample, and internal nodes represent ancestral cells (Beerenwinkel et al, 2014; Schwartz & Schaffer, 2017). Rapid advances in high-throughput next-generation sequencing technologies have made it possible to infer such phylogenies (i.e., cell lineage trees) from a tumor’s mutational landscape. In particular, the recent development of single-cell DNA-sequencing (scDNA-seq) technologies has enabled the identification of individual cancer cell mutations at increasing scale and resolution. Such single-cell resolution data, despite technological limitations such as high error and dropout rates, shows great promise for inferring cell lineage trees and understanding ITH.

Somatic copy number alterations (sCNAs or simply CNAs) are the largest source of genetic heterogeneity in cancer genomes (Beroukhim et al, 2010; Bignell et al, 2010; Zack et al, 2013), making them valuable phylogenetic markers for reconstructing evolutionary trees. However, the properties of CNAs make tree inference challenging; for instance, there is a strong statistical dependence between adjacent genomic loci, and multiple events can overlap the same genomic region. Moreover, there is poor understanding of the distributions that govern CNA rates, sizes, and types (Beerenwinkel et al, 2014; Navin, 2014). Several pioneering studies that leveraged single-cell CNA profiles to build tumor cell lineage trees used traditional correlation distances, such as Euclidean, combined with standard phylogeny inference algorithms (Navin et al, 2011; Wang et al, 2014; Schwartz & Schaffer, 2017; Minussi et al, 2021). Such approaches, however, are thought to be ill-suited for copy number profiles (CNPs) because they make a number of simplifying assumptions and lack a more nuanced probabilistic model of evolutionary distance (Beerenwinkel et al, 2014; Schwartz & Schaffer, 2017), although no systematic evaluation has been performed.

More recently, a number of studies have approached this problem using a framework that explicitly models copy number evolution based on a minimum evolution criterion. These frameworks typically involve finding the minimum event distance (MED), defined as the minimum number of segmental amplifications or deletions needed to transform one genome into another. The MEDICC algorithm was the first method that uses the MED to both phase allele-specific copy numbers and reconstruct a phylogenetic tree from the CNPs (Schwarz et al, 2014). Since then, the MED model has been the focus of numerous other studies. The problem of finding the minimum number of events needed to transform one CNP into another, with certain restrictions, was shown to be solvable in linear time (Zeira et al, 2017), but the original problem approached by MEDICC, which aims to find a tree that minimizes the MED distance globally, was shown to be NP-hard (El-Kebir et al, 2017). There have also been some recent generalizations of the MED model. In the work of Cordonnier and Lafond (2020), individual events can alter copy numbers by any amount and are assigned a positive cost, with the resulting problem being to find a minimum-cost sequence. Zeira and Raphael (2020) proposed weighting events based on their length, event, and type. The MEDALT algorithm (Wang et al, 2021) uses the MED to infer an aneuploidy lineage tree which describes the sequence of events required to evolve one genome into the next, and uses a statistical test to identify CNAs associated with lineage expansion. The MEDICC2 algorithm (Kaufmann et al, 2022) builds upon its predecessor, MEDICC, by explicitly modeling whole-genome duplication (WGD) events and implementing strategies to improve performance. Finally, the Lazac algorithm (Schmidt et al, 2023) solves a relaxation of the small parsimony problem under an approximation of the MED model that allows for the amplification of zero-copy regions.

One of the primary challenges in modeling copy number evolution under the MED framework is its underlying computational complexity. Current MED-based methods do not scale to large datasets, such as those that can be generated by modern scDNA-seq technologies, with many thousands of cells. A few approaches have sidestepped this concern by considering only breakpoints, the genomic locations joining adjacent segments with differing copy numbers. This idea was first developed in the context of bulk-sequencing data to compute clonal relationships between tumor samples of the same patient (Letouze et al, 2010). Breakpoints have also been used in the Bayesian inference procedure of sitka (Salehi et al, 2023), and in an efficient approximation algorithm for the MED problem (Cordonnier & Lafond, 2020). Overall, despite their challenges with scalability, MED-based approaches are currently the gold standard for reconstructing tumor cell lineage trees from single-cell CNA data.

In this work, we introduce two new methods, DICE-bar and DICE-star, based on novel, easy-to-compute distance measures that improve upon the current state-of-the-art in terms of accuracy and scalability. DICE-bar (short for “Distance-based Inference of Copy-number Evolution using breakpoint-root distance”) is a “CNA aware” approach that uses breakpoints between adjacent copy number bins to estimate the number of CNA events. In contrast, DICE-star (short for “Distance-based Inference of Copy-number Evolution using standard-root distance”) uses a simple penalized Manhattan distance between the CNPs themselves. Both methods then use the well-established balanced minimum evolution criterion (Desper & Gascuel, 2002) to reconstruct the final tumor cell lineage tree. Using a large number of realistically simulated datasets, we find that both DICE-bar and DICE-star show strong performance across a wide range of experimental conditions, including different scales, resolutions, noise models, and error rates, while being orders of magnitude faster and more scalable than MED-based methods. Specifically, we find that (i) DICE-bar matches or improves upon the accuracies of all MED-based methods across nearly all tested experimental conditions on both noise-free and noisy data, and (ii) DICE-star further substantially improves upon the accuracies of all existing methods (including DICE-bar), resulting in up to 40% reduction in reconstruction error, on datasets with noise/error levels similar to those observed in real CNPs. Overall, DICE-bar generally matches or exceeds the accuracies of all other methods on noise-free datasets, while DICE-star shows the highest accuracy on datasets with noise/error. These findings hold true even for datasets generated using older, simpler simulators used by existing MED-based methods in their own evaluations.

Our comprehensive experimental analysis identifies DICE-star, given its tolerance to noise, as the most accurate tumor cell lineage tree inference approach for real scDNA-seq-based datasets. Remarkably, DICE-star’s improvements in accuracy over MED-based and other competing methods become even more pronounced as more realistic rates are used for key simulation parameters. Our results also show that DICE-bar, while not as accurate as DICE-star on noisy/error-prone data, provides the best overall performance among all CNA-aware methods, matching or exceeding the accuracies of more complicated MED-based methods. These findings are surprising and significant given the simplicity and scalability of DICE-bar and DICE-star, and since distance-based phylogenetic approaches have traditionally been thought to be ill-suited for CNPs because they do not account for the specific mechanisms of copy number evolution (Beerenwinkel et al, 2014). Our results also clearly demonstrate the effect that noise in CNPs has on the ability of MED-based methods, based on nuanced models of copy number evolution, to effectively reconstruct the underlying phylogeny.

To assess its impact in practice, we applied DICE-star to a number of real scDNA-seq datasets, including 35 breast and ovarian cancer datasets from Funnell et al (2022) and an additional two breast cancer datasets from Navin et al (2011). On the most of these datasets, our analysis reveals that, under multiple metrics, the cell lineage trees computed with DICE-star are either consistent with or more plausible than those of existing methods. These findings highlight the potential real-world impact of the proposed methods. DICE, an umbrella program implementing DICE-star, DICE-bar, and several other distance-based variants, is freely available open-source from https://github.com/samsonweiner/DICE.

## Results

### Overview of DICE-bar and DICE-star

Consistent with other methods, we assume that the evolutionary history of a sampled population of cells can be described by a binary phylogenetic tree, where leaf nodes correspond to the observed single-cell genomes, and internal nodes represent the genomes of ancestral cells. This tree can be rooted along the branch leading to normal (non-cancer) cells or can be left unrooted.

**Figure 1.**
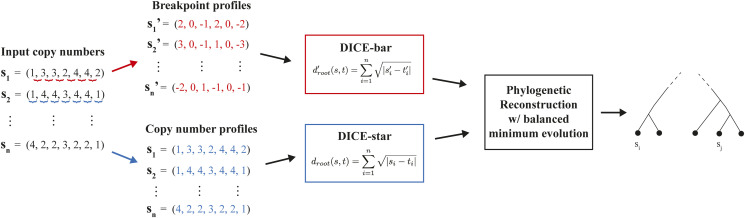
DICE-bar and DICE-star. DICE-bar and DICE-star both reconstruct tumor cell lineages from single-cell copy number profiles provided as input. Both methods employ the “root” distance function as shown, with DICE-star applying it to the CNPs directly (standard variant; shown in blue), and DICE-bar applying it to the relative change in copy number at the breakpoints between adjacent genomic bins (breakpoint variant; shown in red). Once the pairwise distance matrix between cells has been computed, both DICE-star and DICE-bar use the “balanced minimum evolution” criteria to reconstruct a cell lineage tree.

Cells that diverge later during tumor evolution are expected to have many shared alterations present in their genomes, while cells that diverge earlier will have comparatively fewer alterations in common. Thus, the evolutionary relationships among a set of sampled cells can be estimated by comparing the alterations present in their genomes. Given two genomes s and t, we can define a distance function d over their CNPs or breakpoint profiles, such that d*(*s*,*t*)* provides an estimate of the relative evolutionary distance between s and t. Briefly, a CNP is a vector of nonnegative integers describing the copy number of contiguous regions across the genome, and a breakpoint profile is the set of breakpoints for a CNP where a breakpoint is the difference between adjacent copy numbers. DICE-bar and DICE-star are both based on simple, easy-to-compute distance measures to estimate relative evolutionary distances between sampled cells and use an off-the-shelf distance-based phylogeny reconstruction method to reconstruct the final tumor cell lineage tree based on the computed pairwise distances between sampled cells. Fig 1 provides an overview of DICE-bar and DICE-star and shows the key steps in their workflow. As the figure shows, both methods use the same novel distance function but DICE-bar applies this distance function to breakpoint profiles while DICE-star applies it directly to the input CNPs (see the Materials and Methods section for technical details).

### Experimental setup and evaluation metrics

We evaluate the performance of DICE-bar and DICE-star along with eight existing methods, MEDICC2 (Kaufmann et al, 2022), MEDALT (Wang et al, 2021), cnp2cnp (Cordonnier & Lafond, 2020), Lazac (Schmidt et al, 2023), sitka (Salehi et al, 2023), WCND (Zeira & Raphael, 2020), and the distance based approaches of Navin et al (2011) and Minussi et al (2021), using an extensive simulation study encompassing a wide variety of dataset types and conditions. The most of our simulated datasets, consisting of simulated CNPs, were generated using the simulator CNAsim (Weiner & Bansal, 2023a). CNAsim is among the most advanced simulators currently available for simulating single-cell CNPs and implements a broad range of possible CNA mechanisms including WGD, whole-chromosomal CNAs, and chromosome-arm CNAs. CNAsim can also simulate clonal population structure through the accumulation of chromosomal CNAs and implements a realistic error-model that (i) accounts for specific biases of single-cell sequencing that cause fluctuation in read counts, and (ii) models error patterns expected of existing CNA detection algorithms. For additional thoroughness, we also use datasets generated using simpler simulators developed and used by the authors of MEDICC2 (Kaufmann et al, 2022) and cnp2cnp (Cordonnier & Lafond, 2020) to evaluate their own methods. To assess its accuracy and impact in practice, we also apply the best-performing method for noisy datasets, DICE-star, to a number of real breast and ovarian cancer datasets from Funnell et al (2022) and Navin et al (2011). Further details on the simulated and real datasets are available in the Materials and Methods section.

#### Overall experimental setup

We first evaluate DICE-bar, DICE-star, and the eight existing methods using CNAsim datasets simulated under wide range of parameter settings encompassing different experimental conditions, including different numbers of cells, numbers and lengths of chromosomes, CNA rates, copy number bin sizes, WGDs, noise models, and error rates. Second, we use specially generated datasets to assess each method’s ability to accurately detect clones (sometimes also referred to as “subclones” in the literature), i.e., to group cells belonging to the same clone together on the reconstructed cell lineage tree. These special datasets were simulated to contain variable numbers of clones. Third, we evaluate DICE-star, DICE-bar, and the eight other methods on datasets simulated using two previous simulators developed and used in their own evaluation studies by the authors of MEDICC2 (Kaufmann et al, 2022) and cnp2cnp (Cordonnier & Lafond, 2020). We use the same parameter settings used in the original studies to generate these additional datasets, enabling an “apples-to-apples” comparison of the methods, and also explore the impact of using practicable ranges for key simulation parameters. Finally, we apply the best performing method, DICE-star, to publicly available real scDNA-seq datasets from previous studies, analyze the resulting cell lineage trees, and contrast the results against previously reported findings.

#### Evaluation metrics

To evaluate the accuracies of different methods on the simulated datasets, we compare the cell lineage tree reconstructed by each method when applied to a simulated CNP against the known ground truth cell lineage tree used by the simulator for generating that CNP. Specifically, we use the well-known Robinson-Foulds (RF) distance (Robinson & Foulds, 1981) between the reconstructed tree and the corresponding ground truth tree, assuming both trees to be unrooted. Briefly, the RF distance measures the number of bipartitions (or splits) that differ between the two phylogenetic trees being compared. Following standard practice, we normalize the RF distance to be between 0 and 1 by dividing the raw RF distance by the total number of nontrivial bipartitions in the two trees. Thus, a *Normalized RF distance (NRFD)* of 0 indicates the two trees are identical while an NRFD of 1 indicates that the two trees are maximally different (differing in all of their nontrivial bipartitions).

To assess the accuracy of clone detection, we measure how well a reconstructed cell lineage tree identifies each clone (i.e., how well the cells belonging to that clone are grouped together in the reconstructed tree). Specifically, for a given ground truth clone, represented by a subset of cells, we identify the clade/bipartition in the reconstructed tree that shows maximum F1-score with respect to that ground truth clone. We then average these F1-scores across all clones present in the ground truth cell lineage tree. Accordingly, a reported F1 score of 1 implies that all clones were detected with full accuracy. Further information on how ground truth clones are determined appears in the Materials and Methods section.

#### Running existing methods

Of the eight existing methods included in our benchmark, MEDICC2, cnp2cnp, Lazac, and WCND all output binary cell lineage trees with observed cells as leaves. MEDICC2 is also capable of reconstructing ancestral copy number states, in addition to the cell lineage tree; this feature was disabled to enable a fair comparison of running times. For MEDALT and sitka, the output is a nonbinary tree. The MEDALT tree has observed cells as both internal and leaf nodes. To enable an apt comparison, we apply a transformation to the MEDALT tree that ensures each observed sample is represented as a leaf node. The sitka tree outputs observed cells as leaves, so the evaluation metrics can be computed as-is. In addition, because MEDALT, sitka, and cnp2cnp all expect a single copy number at any one locus, allele-specific CNPs were summed together before being passed to them as input. All other methods received allele-specific CNPs as input, whenever available. Finally, the methods of Navin et al (2011) and Minussi et al (2021) do not have associated software implementations and we implemented them ourselves in the DICE software package. Further details on parameter settings and how each method was run appear in the Materials and Methods section.

### DICE-star and DICE-bar outperform other methods

We first used our baseline datasets, simulated using CNAsim with the default parameter values and three different noise levels (no noise, low noise, and high noise; see the Materials and Methods section for details), and additional datasets aimed at assessing the ability of the different methods to accurately detect clones, to assess all 10 methods. The error-rates for low-noise and high-noise datasets were selected to match the breakpoint detection accuracy of existing CNA detection algorithms (Mallory et al, 2020a), with low-noise datasets corresponding to the higher end of observed precision and recall values and high-noise datasets corresponding to the middle of observed ranges for precision and recall. Further details on error rates and noise parameters appear in the Materials and Methods section. No-noise datasets represent the ideal case when all copy number alterations are inferred without any error.

#### Cell lineage reconstruction accuracy

**Figure 2.**
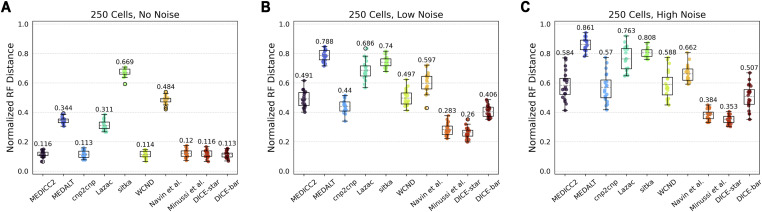
Cell lineage tree reconstruction accuracies. Box and whisker plots are shown for DICE-bar, DICE-star, and eight existing methods on simulated datasets with 250 cells and varying levels of noise. **(A, B, C)** Results are shown for datasets with: (A) no noise; (B) low noise; (C) high noise. Lower normalized RF Distances imply greater reconstruction accuracy. Data were generated using CNAsim and default simulation settings, and error rates for low and high noise levels were selected to match the precision and recall characteristics of breakpoint detection from existing CNA detection algorithms. Observe that DICE-bar matches or improves upon the accuracies of all other methods on noise-free datasets and DICE-star improves upon the accuracies of all other methods on the noisy datasets.

Fig 2 shows the accuracies of cell lineage reconstruction for all 10 methods on the baseline datasets. For each of the three noise levels, reported accuracies are averaged over 20 datasets, where each dataset consists of 250 cells and uses 22 chromosomes with lengths based on the human reference genome hg38 (and default values for all other simulation parameters). As expected, we find that the presence of noise results in higher mean reconstruction error and overall variance for all methods. This analysis reveals important insights into the relative performance (accuracies) of the different methods. In particular, we find that (i) DICE-bar matches or improves upon the accuracies of all existing methods on the noise-free datasets, and improves upon the accuracies of the more sophisticated methods MEDICC2, MEDALT, cnp2cnp, Lazac, and WCND even on the noisy datasets, and (ii) DICE-star shows exceptional robustness to noise and improves upon the accuracies of all existing methods, and of DICE-bar, on all noisy datasets. For example, on the high-noise datasets, DICE-star and DICE-bar show NRFDs of 0.353 and 0.507, respectively, while the existing methods MEDICC2, MEDALT, cnp2cnp, Lazac, Sitka, WCND, Navin et al, and Minussi et al show NRFDs of 0.584, 0.861, 0.57, 0.763, 0.808, 0.588, 0.662, and 0.384, respectively. Remarkably, DICE-star improves upon the NRFDs of the top performing MED-based methods MEDICC2 and cnp2cnp by an average of 43% and 39.3%, respectively, for the noisy datasets.

More generally, these results suggest that methods based on MED or breakpoint distances, though accurate on noise-free data, can be highly sensitive to error and noise in inferred CNPs. Likewise, these results also show that methods based on appropriately designed distance measures between CNPs, such as DICE-star and the method of Minussi et al (which uses standard Manhattan distance between CNPs), though not as accurate as the best MED or breakpoint methods on noise-free data, are far more robust to realistic levels of noise and error in CNPs. These findings have important implications for the application of these methods to real datasets.

**Figure S1.**
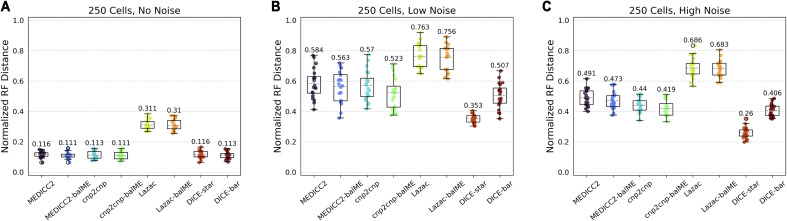
Reconstruction accuracies of existing methods when using balanced minimum evolution for tree inference. Cell lineage tree reconstruction accuracies are shown for MEDICC2, cnp2cnp, and Lazac when using their respective builtin (default) tree reconstruction algorithms and when applying the balanced minimum-evolution tree reconstruction algorithm to the pairwise distances computed under each respective model. Results for DICE-star and DICE-bar are also shown for comparison. The datasets correspond to those appearing in Fig 2 of the main text. **(A, B, C)** Results are shown for datasets with: (A) no noise; (B) low noise; (C) high noise. All results are averaged over 20 simulated datasets.

For completeness, we also investigated if the improved cell lineage tree reconstruction accuracies of DICE-bar and DICE-star are a result of the distance measures used or of the specific distance-based phylogenetic reconstruction algorithm employed (balanced minimum evolution). Accordingly, we applied balanced minimum evolution to distance matrices computed under the respective models of MEDICC2, cnp2cnp, and Lazac. These results are shown in Fig S1. We find that using balanced minimum evolution leads to a slight improvement in accuracy for each method compared with their respective built-in approaches. However, as Fig S1 shows, DICE-bar continues to generally match or outperform all methods for all noise levels and DICE-star continues to substantially outperform all methods on the noisy datasets. This shows that the improvements offered by DICE-bar and DICE-star result primarily from the distance measure themselves.

#### Clone detection accuracy

**Figure 3.**
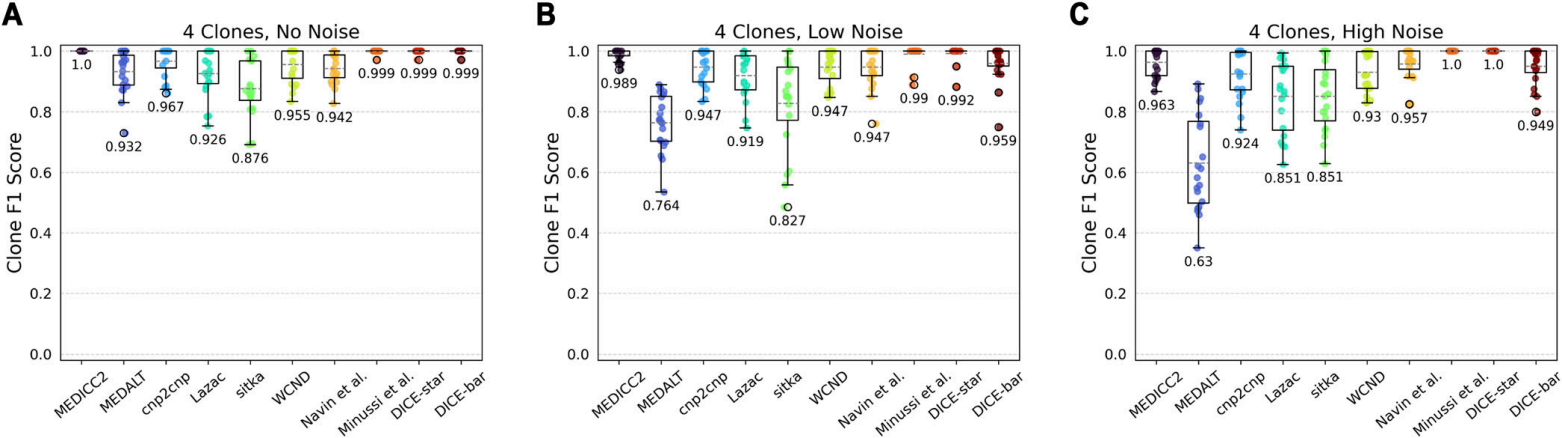
Clone detection accuracies. Box and whisker plots of F1 scores are shown for DICE-bar, DICE-star, and eight existing methods on simulated datasets with 250 cells and varying levels of noise. Scores are averaged over 20 datasets, each with exactly four clones. **(A, B, C)** Results are shown for datasets with: (A) no noise; (B) low noise; (C) high noise. Higher F1 scores are better. As the plot shows, DICE-bar, DICE-star, MEDICC2, and Minussi et al show perfect or near-perfect F1 scores on noise-free data, while DICE-star, Minussi et al, and MEDICC2 exceed the F1 scores of all other methods on the noisy datasets.

To evaluate clone detection, we generated datasets containing a variable number of clones in the ground truth tree, with all other parameters kept at default values. Fig 3 shows the results of DICE-bar, DICE-star, and existing methods on trees containing exactly four clones. On noise-free data, we find that most methods perform very well at capturing clonal populations. In particular, both DICE-bar and DICE-star and MEDICC2 and the method of Minussi et al obtain perfect or near-perfect scores. On noisy data, most methods show a decrease in performance, with DICE-bar and the MED-based methods MEDICC2, MEDALT, cnp2cnp, Lazac, and WCND all showing at least a slight drop in clone detection accuracy. However, DICE-star and the method of Minussi et al show greater robustness to noise, yielding higher F1 scores than the other methods at both low and high levels of noise. For example, both DICE-star and the method of Minussi et al show an F1 score of 1.0 on the high-noise datasets, while MEDICC2, MEDALT, cnp2cnp, Lazac, sitka, WCND, Navin et al, and DICE-bar show F1 scores of 0.963, 0.63, 0.924, 0.851, 0.851, 0.93, 0.957, and 0.949, respectively. Among CNA-aware methods, MEDICC2 shows greater robustness to noise than the other MED- or breakpoint-based methods. Sitka also appears to be robust to noise, likely due to its loci preprocessing step, but has an overall poor baseline performance compared with the other methods. Overall, these results further support the suitability of DICE-star for real datasets.

**Figure S2.**
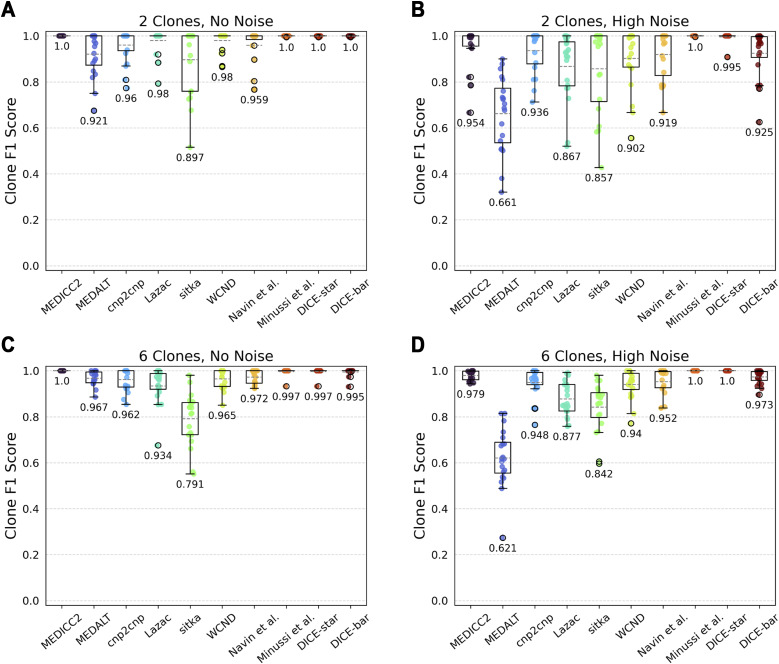
Clone detection accuracies on datasets with varying numbers of clones. (A, B, C, D) F1 scores are shown for the different methods on simulated datasets with 250 cells and either no noise (left column; (A, C)) or high noise (right column; (B, D)). Scores are averaged over 20 datasets, each with exactly two (top row; (A, B)) or exactly six (bottom row; (C, D)) clones. Observe that these results are consistent with those shown in Fig 3 in the main text which shows results for exactly four clones.

**Figure S3.**
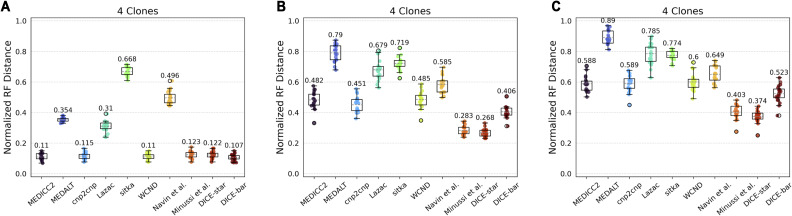
Cell lineage tree reconstruction accuracy on four-clone trees. Cell lineage tree reconstruction accuracies are shown for the datasets used in Fig 3 of the main text. **(A, B, C)** Results for datasets with: (A) no noise; (B) low noise; (C) high noise. Observe that performance across all methods is largely unaffected by the presence of clonal structures, with DICE-bar continuing to match or outperform all other methods on noise-free datasets and DICE-star continuing to outperform all other methods on the low- and high-noise datasets. Scores are averaged over 20 datasets, each with exactly four clones.

Importantly, we found that clone detection performance remains virtually unchanged when varying the number of clones (Fig S2). We also note that the presence of clones has little to no effect on overall cell lineage reconstruction accuracy (Fig S3).

### DICE-star and DICE-bar are robust to evolutionary and experimental conditions

Next, we evaluated the methods on datasets representing different evolutionary and experimental conditions such as different numbers of cells, genome/chromosome sizes, CNA rates, bin sizes, etc. For each condition, we varied the relevant simulation parameter, keeping other parameters at their default values, and generated no-noise and high-noise datasets.

#### Number of cells

**Figure S4.**
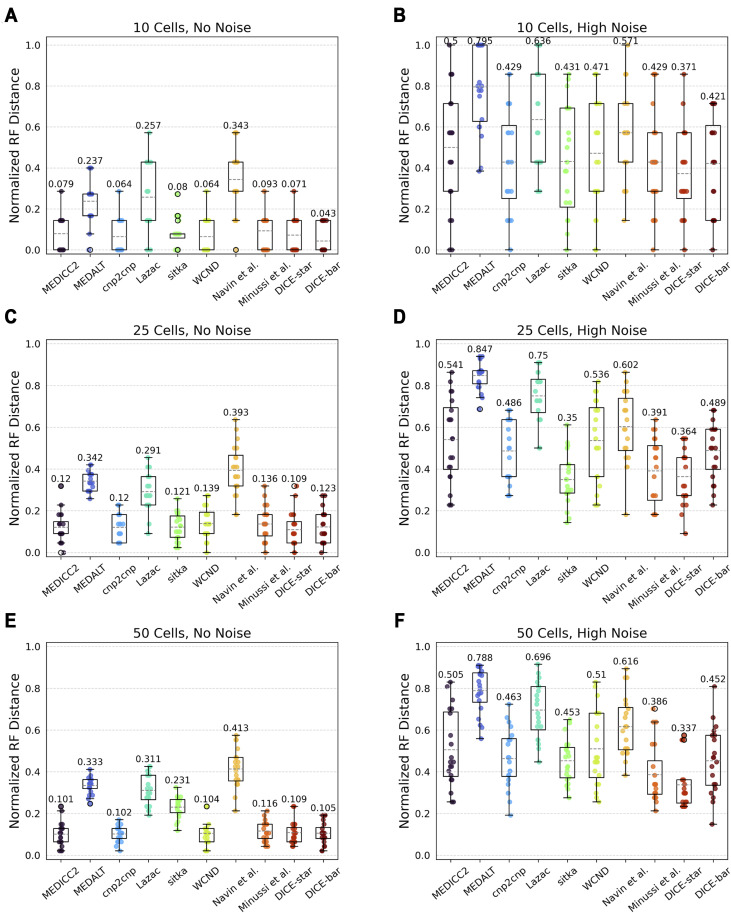
Reconstruction accuracy for smaller numbers of cells. (A, B, C, D, E, F) Cell lineage tree reconstruction accuracies are shown for different methods on simulated datasets with varying numbers of cells (rows; (A, B): 10 cells, (C, D): 25 cells, and (E, F): 50 cells) and at two different noise levels (columns; (A, C, E): no-noise and (B, D, F): high-noise). Lower normalized RF distances imply greater tree reconstruction accuracy. All reported results are averaged over 20 datasets.

**Figure S5.**
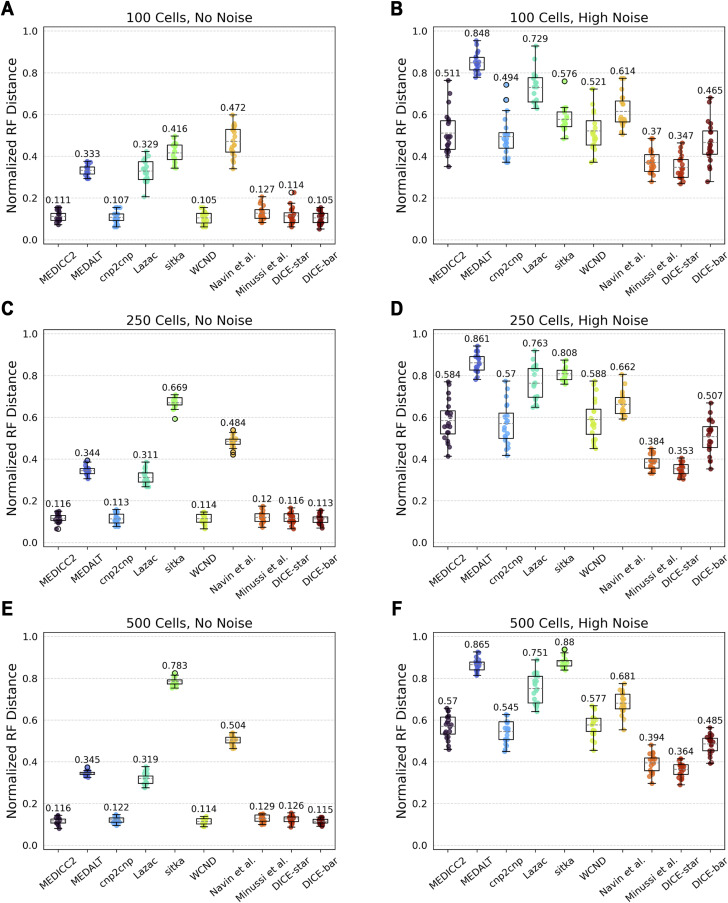
Reconstruction accuracy for larger numbers of cells. **(A, B, C, D, E, F)** Cell lineage tree reconstruction accuracies are shown for different methods on simulated datasets with varying numbers of cells (rows; (A, B): 100 cells, (C, D): 250 cells, and (E, F): 500 cells) and at two different noise levels (columns; (A, C, E): no-noise and (B, D, F): high-noise). Lower normalized RF distances imply greater tree reconstruction accuracy. All reported results are averaged over 20 datasets.

We evaluated the impact of number of cells (i.e., tree size) using datasets with 10, 25, 50, 100, 250, and 500 cells. Figs S4 and S5 show the results of this analysis. Consistent with previous results, we find that DICE-bar generally matches or exceeds the accuracies of all other methods on all noise-free datasets except for the one with 25 cells where, unexpectedly, DICE-star becomes the best performing method. MEDICC2, cnp2cnp, and WCND also perform well on the noise-free datasets, generally matching the accuracy of DICE-bar on several of these datasets. For the noisy datasets, we again find that DICE-star outperforms the other methods, including DICE-bar. Overall, we find that the accuracy of most methods is only slightly affected by the number of cells but that variance across replicates consistently decreases with increasing numbers of cells. The one exception to this is sitka, which achieves competitive performance on small trees only and, interestingly, shows robustness to noise, even slightly outperforming DICE-star in the 25-cell setting. This is likely due to the highly selective loci filtering step employed by sitka, which greatly restricts the number of loci used for phylogenetic reconstruction. The small number of filtered loci is only sufficient for reconstructing trees for small numbers of cells.

#### Genome size

**Figure S6.**
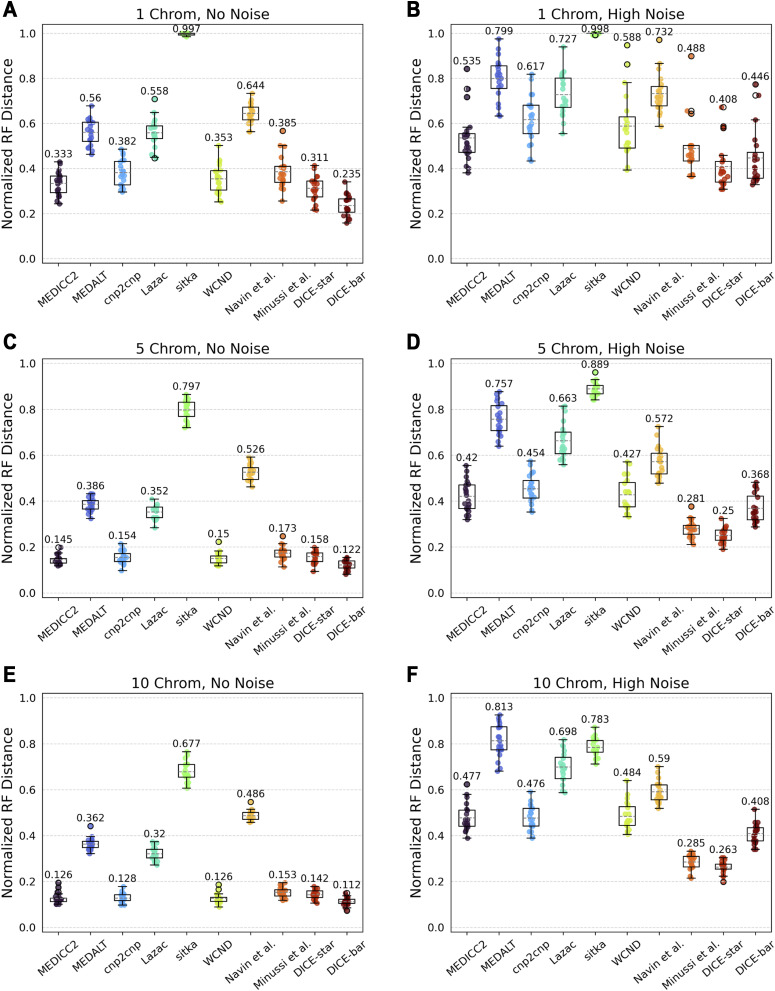
Impact of number of chromosomes on reconstruction accuracy. **(A, B, C, D, E, F)** Cell lineage tree reconstruction accuracies are shown for different methods on simulated datasets with varying numbers of chromosomes (rows; (A, B): 1, (C, D): 5, and (E, F): 10 chromosomes) and at two different noise levels (columns; (A, C, E): no-noise and (B, D, F): high-noise). Lower normalized RF distances imply greater tree reconstruction accuracy. All reported results are averaged over 20 datasets.

**Figure S7.**
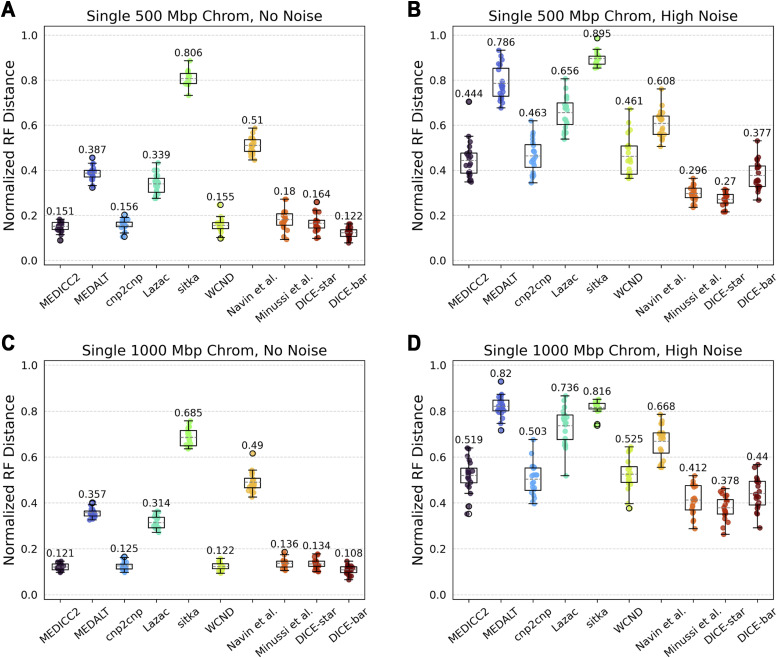
Reconstruction accuracy on single chromosomes of variable length. **(A, B, C, D)** Cell lineage tree reconstruction accuracies are shown for different methods on simulated datasets with chromosomes of variable length (rows; (A, B): 500 and (C, D): 1,000 Mbp chromosome lengths) and at two different noise levels (columns; (A, C): no-noise and (B, D): high-noise). Lower normalized RF distances imply greater tree reconstruction accuracy. All reported results are averaged over 20 datasets.

We next evaluated the impact of genome size by generating datasets with varying number of chromosomes, each with a length of 100 Mbp, and keeping all other parameters at default values. Fig S6 shows the results of this analysis. On the noise-free datasets, we find that all methods perform worse with fewer chromosomes and that DICE-bar outperforms all other methods. On the noisy datasets, DICE-star outperforms all other methods for all numbers of chromosomes. We also find that the performance of all methods slightly worsens as the number of chromosomes increases from 5 to 10 for the noisy datasets. This may be an artifact of the error model used in CNAsim, or because the methods see no additional benefit from additional error-prone CNPs beyond five chromosomes given that the number of cells in these datasets is only 250. For completeness, we also performed an equivalent experiment where the number of chromosomes was fixed at 1 but chromosome length varied; for example, five chromosomes with length 100 Mbp would correspond to 1 chromosome with length 500 Mbp. We found nearly identical results across the two sets of experiments (Fig S7), suggesting that the total amount of available genomic information is the more impactful factor, regardless of how it is distributed across chromosomes.

#### Presence of WGD

**Figure S8.**
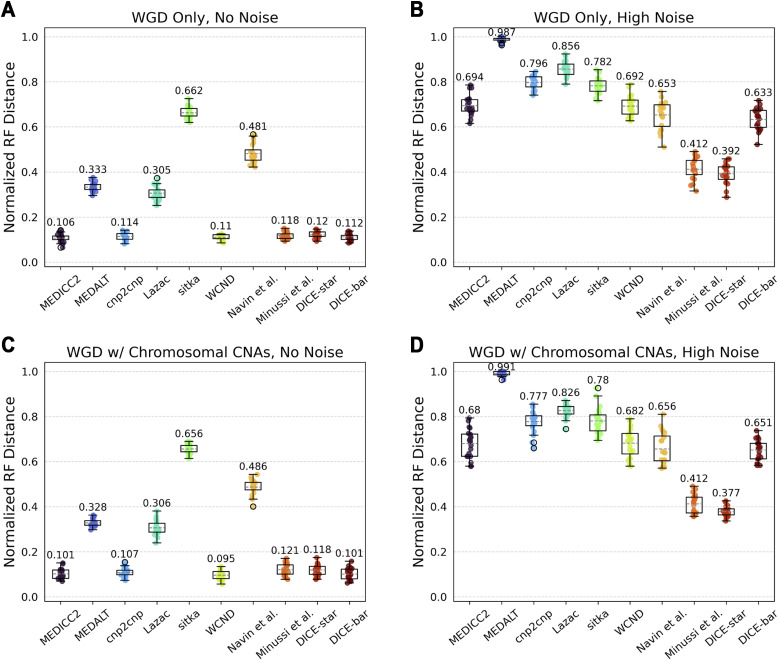
Reconstruction accuracy in the presence of WGD. Cell lineage tree reconstruction accuracies are shown for different methods on simulated datasets with WGD (rows) and at two different noise levels (columns; no-noise and high-noise). **(A, B, C, D)** WGD was evaluated both individually (A, B) and in the presence of chromosomal CNAs (C, D). For the latter, a mean of two chromosomal events occur on the edges into and out of the root node, and into four clonal clades existing in the phylogeny. Lower normalized RF distances imply greater tree reconstruction accuracy. On noise-free data, performance slightly improves when chromosomal CNAs are included but is otherwise unaffected by WGD. On noisy data, Minussi et al and DICE-star perform slightly worse in the presence of WGD, while all other methods suffer heavily from the increase in ploidy due to WGD. All reported results are averaged over 20 datasets.

We find that the presence of WGD has little impact on reconstruction accuracy in the noise-free setting, but that all methods show worse performance with WGD (than without WGD) on the noisy datasets (Fig S8). In addition, as the figure shows, most methods appear to improve slightly if the WGD is followed by chromosomal CNAs. Overall, DICE-star remains the best performing method on the noisy datasets, while DICE-bar, WCND, cnp2cnp, and MEDICC2 generally match or exceed the accuracies of the other methods on noise-free datasets. Interestingly, MEDICC2 becomes the best performing method if there is no noise and the WGD is introduced as the sole large-scale copy number event, likely because MEDICC2 is the only method that makes an explicit attempt to model WGD. Still, DICE-star outperforms MEDICC2 on all noisy datasets, cutting its reconstruction error almost in half.

#### Number of CNA events

**Figure S9.**
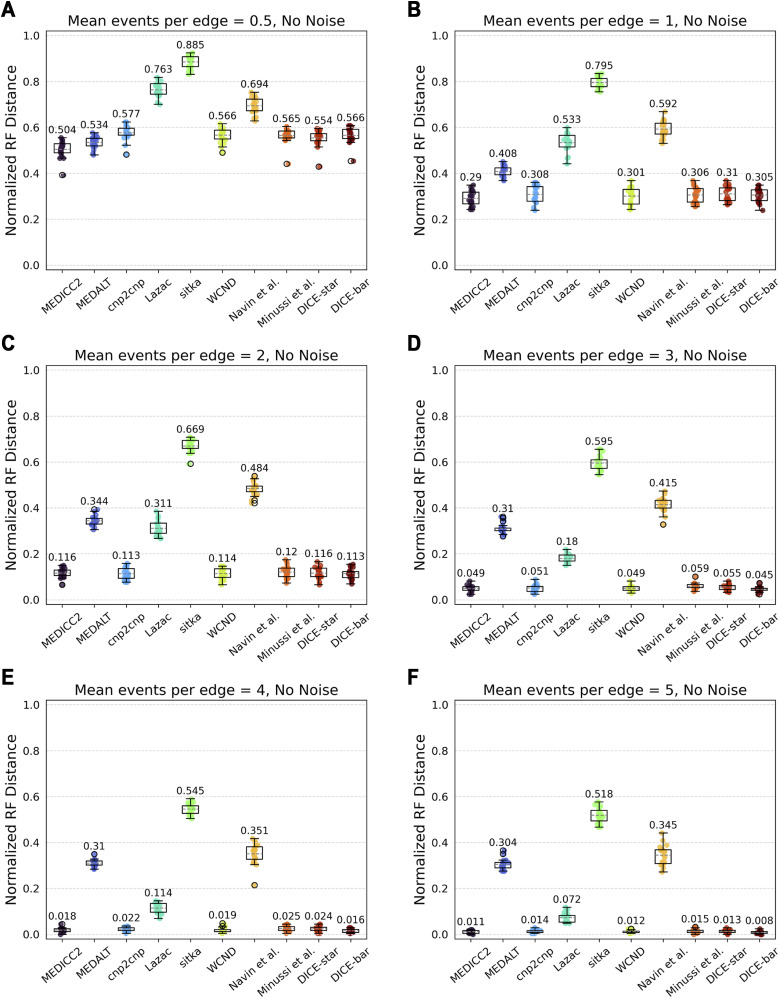
Impact of events per edge on reconstruction accuracy using noise-free data. **(A, B, C, D, E, F)** Cell lineage tree reconstruction accuracies are shown for different methods on simulated noise-free datasets with increasing numbers of focal CNAs per edge ((A, B, C, D, E, F) with 0.5, 1, 2, 3, 4, and 5 events per edge, respectively). Lower normalized RF distances imply greater tree reconstruction accuracy. For every one additional event per edge, the normalized RF distance roughly halves for all methods except MEDALT, Sitka, and Navin et al. All reported results are averaged over 20 datasets.

**Figure S10.**
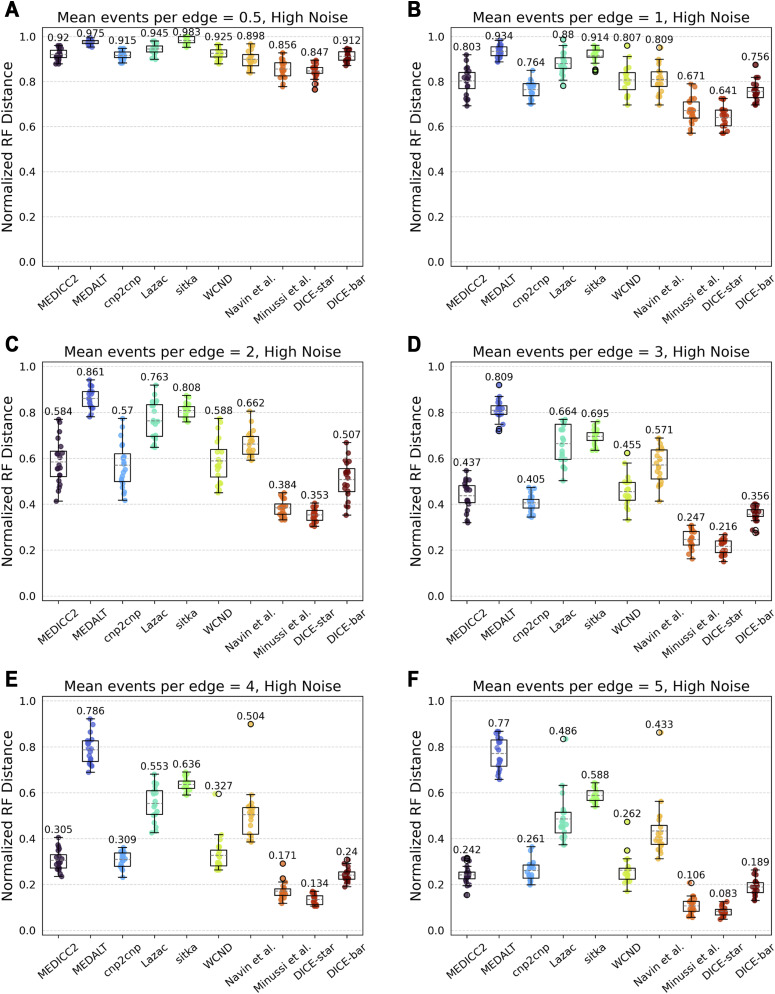
Impact of events per edge on reconstruction accuracy using noisy data. **(A, B, C, D, E, F)** Cell lineage tree reconstruction accuracies are shown for different methods on simulated noisy datasets with increasing numbers of focal CNAs per edge ((A, B, C, D, E, F) with 0.5, 1, 2, 3, 4, and 5 events per edge, respectively). Lower normalized RF distances imply greater tree reconstruction accuracy. These datasets correspond to those depicted in Figure 16 using a high noise rate. Increasing the mutation rate substantially improves the performance of all methods except for MEDALT and Sitka, which only improve slightly. **(A, B)** Observe that the effect of noise is amplified under very low mutations rates, as the CNA-to-noise ratio is severely imbalanced. All reported results are averaged over 20 datasets.

We varied the mean number of segmental CNAs per edge (parameter λ) in the range 0.5–5 and found it to be among the most impactful parameters for cell lineage reconstruction accuracy. As Figs S9 and S10 show, increasing CNA event rates lead to significant improvements in reconstruction accuracies for all methods. On noise-free datasets, MEDICC2, cnp2cnp, WCND, Minussi et al, DICE-star, and DICE-bar all achieve near-perfect reconstruction accuracy when λ is set to 5 (Fig S9). This is likely because the abundance of mutations along each edge substantially reduces the uncertainty of evolutionary relationships. On noise-free datasets, MEDICC2 outperforms all other methods for the two lowest values of λ, with DICE-bar matching or exceeding the accuracies of other methods on all other noise-free datasets. This indicates that the MED model is best suited for low mutation rates. However, as expected, the performance of MEDICC2 falls sharply on noisy datasets, and DICE-star again shows the best performance across all CNA event rates on noisy datasets (Fig S10).

#### Number of bins and bin size

**Figure S11.**
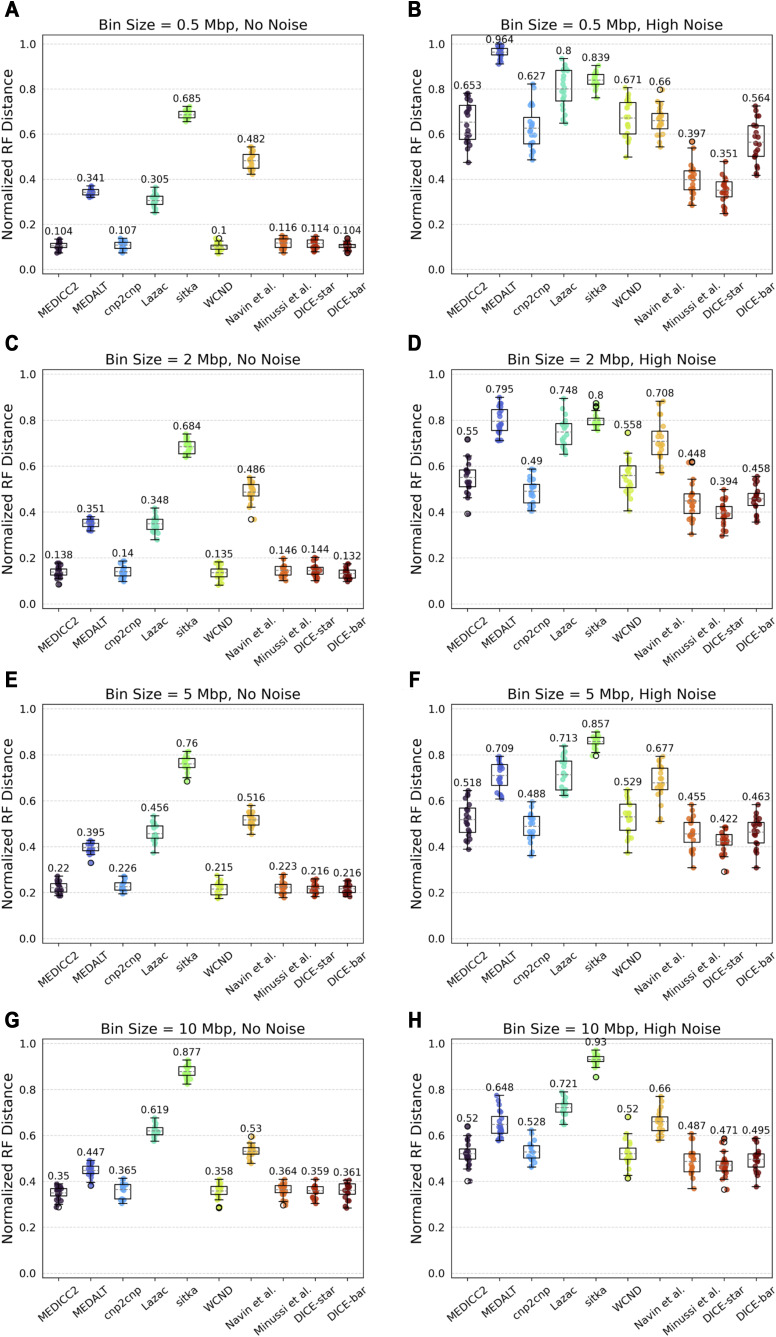
Impact of bin size on reconstruction accuracy. **(A, B, C, D, E, F, G, H)** Cell lineage reconstruction accuracies are shown for different methods on simulated datasets with increasing bin size (rows; (A, B): 0.5, (C, D): 2, (E, F): 5, and (G, H): 10 Mbp bin sizes) and at two different noise levels (columns; (A, C, E, G): no-noise and (B, D, F, H): high-noise). Lower normalized RF distances imply greater tree reconstruction accuracy. On noise-free datasets, across all methods, performance steadily decreases with an increase in bin size. This trend is, surprisingly, large reversed on noisy datasets, with only DICE-star and Minussi et al clearly benefiting from smaller bin sizes. All reported results are averaged over 20 datasets.

Finally, we evaluated the impact of bin size (or, equivalently, number of bins) by considering four different bin sizes in the range 0.5–10 Mbp. Results are shown in Fig S11. On the noise-free datasets, we find that accuracy steadily decreases with increasing bin size. This is expected because smaller bin sizes (which leads to a greater number of total bins) provide more information for phylogenetic reconstruction. However, we see mixed results on the noisy datasets, with only DICE-star and Minussi et al clearly benefiting from smaller bin sizes. This suggests that the other methods, which are all more susceptible to noise, may be unable to benefit from smaller bin sizes on real, error-prone datasets. DICE-star remains the best method on noisy datasets across all bin sizes, and DICE-bar matches or slightly outperforms the existing methods on noise-free datasets for three of the four bin-sizes. On the noise-free dataset with largest bin-size, MEDICC2 shows slightly better accuracy than DICE-bar.

Overall, the above results demonstrate the robustness of DICE-star and DICE-bar to different evolutionary and experimental conditions and further establish DICE-bar as among the best methods to use for noise-free data and DICE-star as the best method to use for noisy data. These results also suggest that MEDICC2 does well in scenarios where there is limited information in the CNPs, e.g., when the rate of events is low or when bin sizes are very large, as long as the CNPs are noise-free.

### DICE-star is robust to CNA estimation error

To better understand the effect of CNA estimation error (i.e., noise in input CNPs) on cell lineage reconstruction accuracy, we generated datasets using several additional combinations of the noise parameters. Specifically, we fixed the jitter error rate at two values, 0.05 and 0.15, representing a low and high baseline rate, and varied the boundary error rate from a low of 0.02 to a high of 0.14 at intervals of 0.02. For reference, Table S1 shows how these error rates affect the precision and recall of ground truth CNPs.

**Figure 4.**
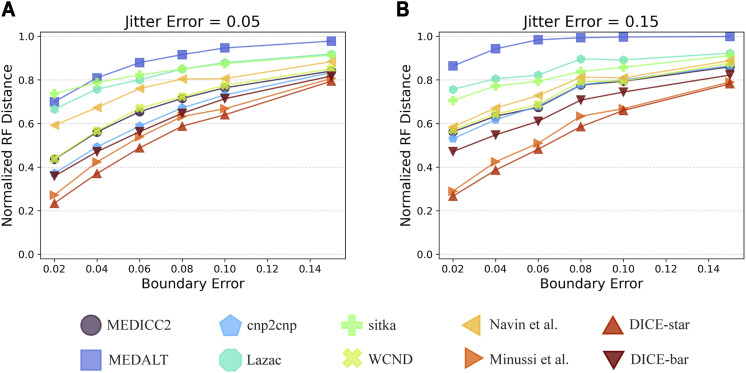
Impact of CNA estimation error on the different methods. The plots show how the individual error models (boundary and jitter) and their error rates affect cell lineage reconstruction accuracy. The parameter of the jitter model was fixed and various parameters were evaluated for the boundary model (y-axis). **(A, B)** The jitter model was fixed at a “low” error rate with *r*_*j*_ *= 0.05* (A) and a “high” error rate with *r*_*j*_ *= 0.15* (B). For each jitter error rate, the plots show reconstruction accuracy results for seven different boundary error rates. Observe that DICE-star outperforms all other methods for all error-rates, and that even DICE-bar matches or outperforms the more sophisticated methods MEDICC2, MEDALT, cnp2cnp, Lazac, sitka, and WCND across all error-rates.

Table S1. Precision and recall values for various combinations of noise parameters. The reported precision and recall values are estimated by comparing ground truth CNPs with the generated, noisy CNPs. Datasets A1–A6 correspond to those depicted in Fig 4A, while datasets B1–B6 correspond to those depicted in Fig 4B. All reported values are averaged over 20 datasets.

Fig 4 shows the results of applying all 10 methods to these datasets. As the figure show, the performance of all methods degrades rapidly as the boundary error-rate increases. More importantly, regardless of the source of noise (i.e., the boundary model or jitter model), DICE-star outperforms all other methods at all noise levels. This improvement over other methods is greatest when the rate of jitter error is high, but the magnitude of improvement decreases with increasing boundary error rate. Interestingly, while MEDICC2, MEDALT, cnp2cnp, Lazac, WCND, and DICE-bar are all substantially negatively impacted by increased jitter, this is not the case for DICE-star and the method of Minussi et al (2021). We also note that DICE-bar matches or improves upon the accuracies of MEDICC2, MEDALT, cnp2cnp, Lazac, sitka, and WCND across all error rates.

### Other simulators support strong performance of DICE-bar and DICE-star

#### MEDICC2 simulator

**Figure S12.**
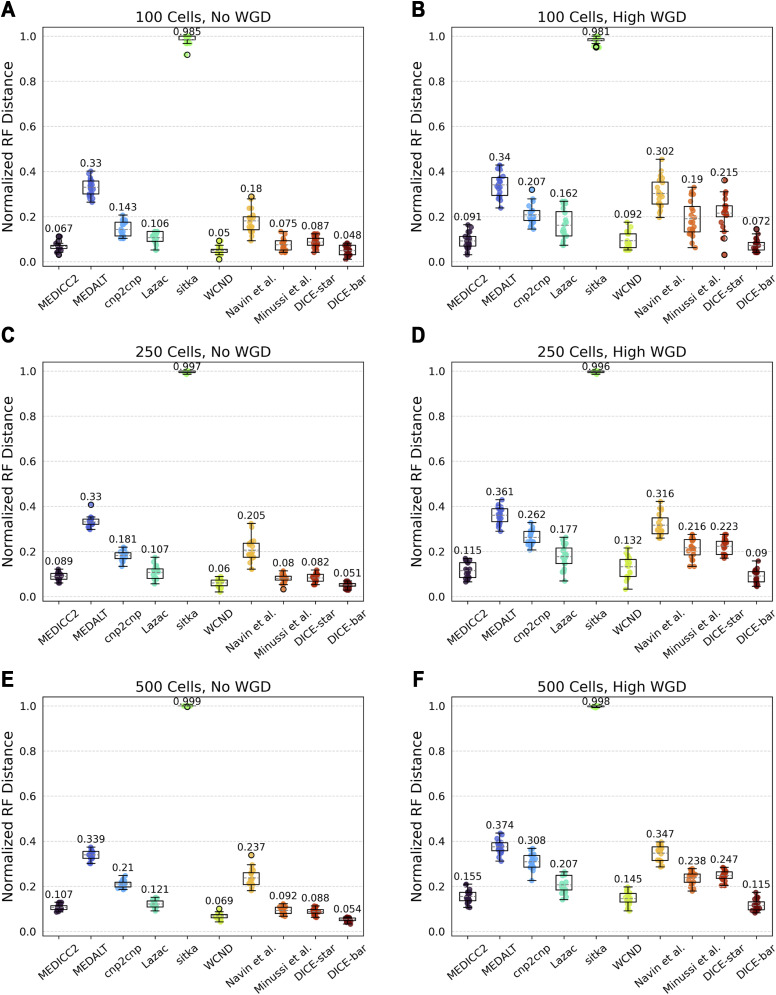
Results using MEDICC2’s simulation framework with default parameters. Cell lineage reconstruction accuracies are shown for different methods on datasets simulated using the simulation routine of MEDICC2 with default parameters and no noise. Left column: (A, C, E) results on datasets with WGD rate of 0 and an increasing number of cells. Right column: (B, D, F) Results on datasets with high WGD rate and increasing number of cells. Simulation parameter values were selected as those described in Kaufmann et al (2022). The results show similar trends as those on noise-free simulated datasets of CNAsim, with DICE-bar outperforming all other methods.

We generated datasets of various sizes using the MEDICC2 simulator with its default parameters and with their most frequently used mutation rate of 0.05 (Kaufmann et al, 2022). These datasets consist of three sizes (100, 250, and 500 cells), and for each size includes datasets with no WGD and a high rate of WGD. We note that the MEDICC2 simulator cannot simulate noisy CNPs, and therefore all MEDICC2 datasets are noise-free. Fig S12 shows the results of applying all 10 methods to these datasets. Consistent with our previous results on noise-free datasets, DICE-bar shows higher accuracy than all other methods on both the no-WGD and high-WGD datasets. WCND and MEDICC2 also perform well on both the no-WGD and high-WGD datasets, while DICE-star and the method of Minussi et al (2021) perform well on the no-WGD datasets. Interestingly, the presence of WGD appears to have a substantial negative impact on the performance of all methods, although MEDICC2, WCND, and DICE-bar appear to be less affected than other methods. We also note that sitka performs extremely poorly on these datasets, likely due to its strict loci filtering step. Overall, these results identify DICE-bar as the most accurate method on noise-free data.

**Figure S13.**
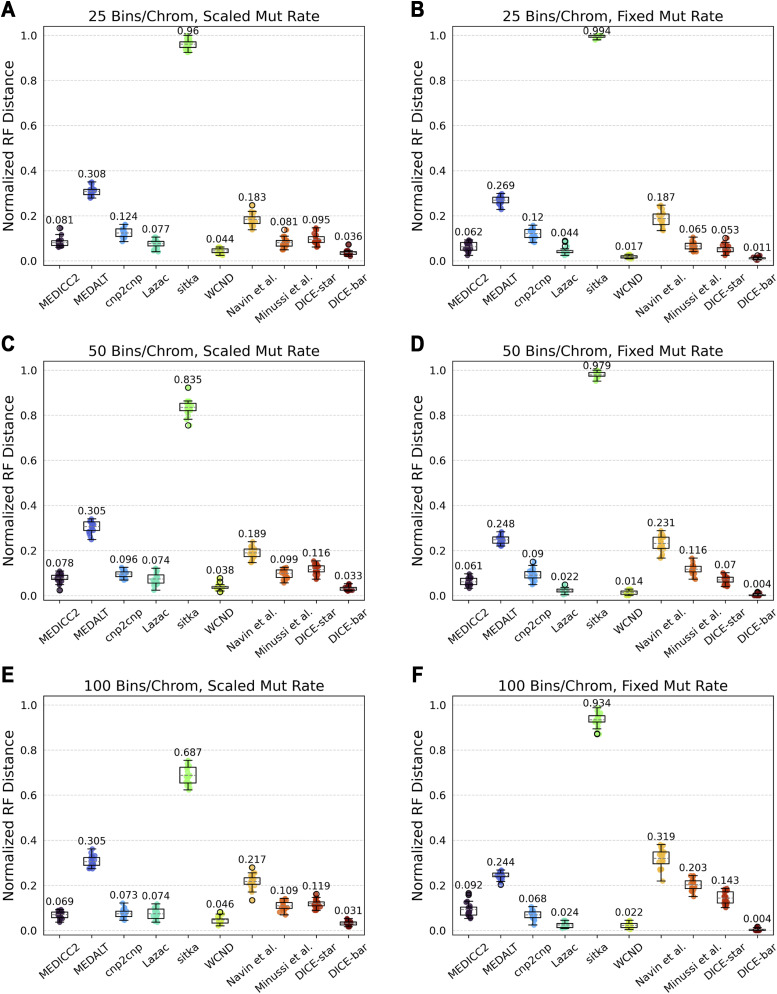
Results using MEDICC2’s simulation framework with increasing number of bins per chromosome. Cell lineage reconstruction accuracies are shown for different methods on datasets simulated using the simulation routine of MEDICC2 with default parameters, no noise, and increasing numbers of bins (rows; 25, 50, and 100 bins per chromosome). Left column: (A, C, E) Performance on simulated datasets of increasing number of bins per chromosome with a scaled mutation rate such that the number of events per edge is consistent across all settings. Right column: (B, D, F) Performance on simulated datasets of increasing number of bins per chromosome with a fixed mutation rate of 0.05. Note that the number of events per edge in the MEDICC2 simulation routine scales with total genome size, so datasets of varying genome size cannot be consistently compared when using a fixed mutation rate. Observe that DICE-bar outperforms all other methods on these datasets. All reported results are averaged over 20 datasets.

For greater realism, we also used the MEDICC2 simulator to generate datasets with additional bins per chromosome. At 22 chromosomes, the MEDICC2 simulator by default only uses 10 bins per chromosome for a total of 2 × 22 × 10 = 440, which may not be realistic for whole-genome datasets. For reference, the default CNAsim parameters of using 1 Mbp bin sizes over the 22 autosome lengths derived from hg38 equates to over 5,700 bins. Because the MEDICC2 simulator uses a number of mutations that scales linearly with the total number of bins, we used a scaled mutation rate to maintain consistency with default settings; however, for comparison we also show results using a fixed mutation rate. Results are shown in Fig S13. We find that DICE-bar continues to be the best performing method, improving upon the accuracy of the nearest competitor, WCND, by at least 15%. Interestingly, while MEDICC2, cnp2cnp, sitka, and DICE-bar all show a steady improvement in performance as the number of bins increases with scaled mutation rates, the performance of the remaining methods remains relatively unchanged or slightly worsens. In contrast, genome size strongly correlated with performance across all methods in the CNAsim datasets (Figs S6 and S7). When keeping the mutation rate fixed, which effectively increases the number of mutations, DICE-bar achieves near perfect reconstruction accuracy; however, the other methods either show similar results to the scaled setting or become worse. This result is unexpected, as other methods do benefit from an increased mutation rate in the CNAsim datasets (Fig S9E and F). This discrepancy can likely be attributed to the many types of mutations outside of segmental duplications and deletions implemented in the MEDICC2 simulator.

#### cnp2cnp simulator

**Figure S14.**
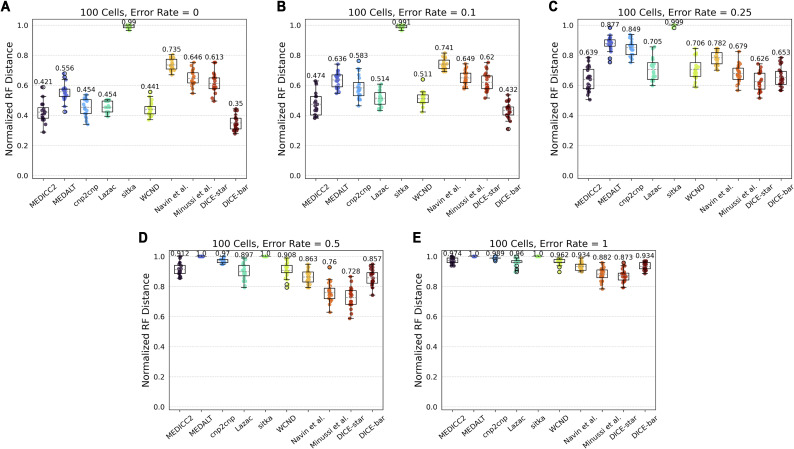
Results using cnp2cnp′s simulation framework with default parameters. **(A, B, C, D, E)** Cell lineage reconstruction accuracies are shown for different methods on 100-cell datasets simulated using the simulation routine of cnp2cnp with default parameters and increasing levels of noise ((A, B, C, D, E) with error rates 0, 0.1, 0.25, 0.5, and 1, respectively). Observe that the performance of all methods is significantly worse compared with performance on the simulated datasets of CNAsim, likely due to the very low default number of bins (set to 100 on a single chromosome). DICE-bar outperforms all other methods for the two lowest noise levels, while DICE-star outperforms all other methods for all remaining (higher) noise levels. All results are averaged over 20 datasets.

Next, we evaluated the different methods on 100-cell datasets generated with the cnp2cnp simulator using its default parameter values. This cnp2cnp simulator implements a simple error model to generate noisy CNPs, and Fig S14 shows results on datasets with increasing levels of noise. As the figure shows, DICE-bar outperforms all other methods on the two lowest noise datasets, while DICE-star begins to significantly outperform all other methods for the remaining three datasets with higher noise levels. These results are fully consistent with our previous findings. We also find that the performance of all methods is worse on the cnp2cnp datasets than on the baseline CNAsim datasets. This is likely due to the very low default number of bins, set to 100 on a single chromosome, in the cnp2cnp simulator.

**Figure S15.**
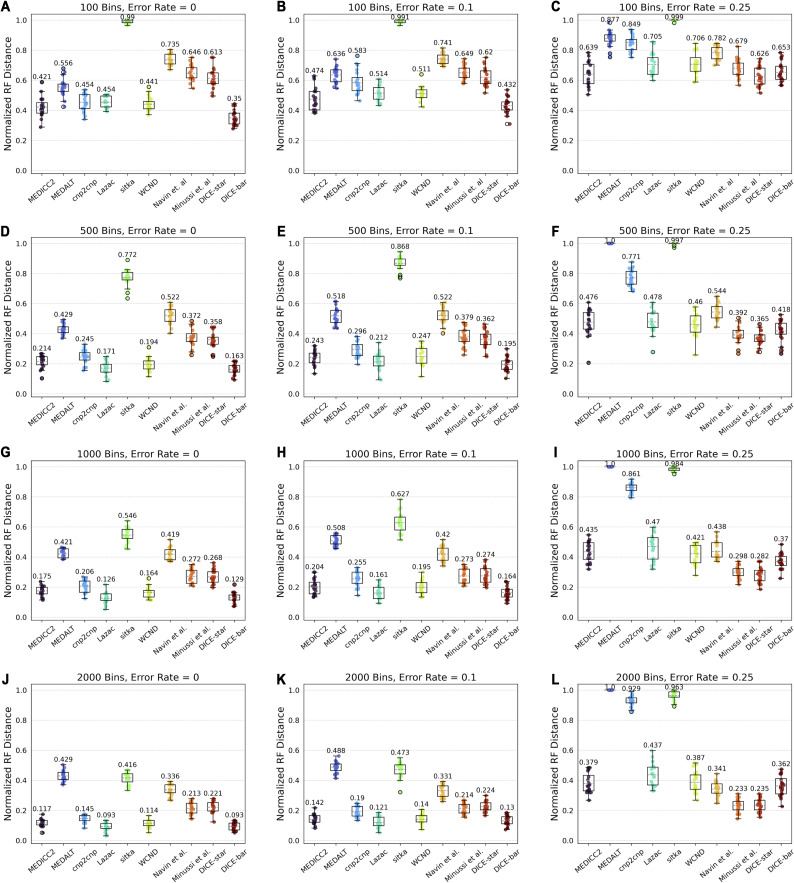
Results using cnp2cnp′s simulation framework with increasing number of bins. **(A, B, C, D, E, F, G, H, I, J, K, L)** Cell lineage reconstruction accuracies are shown for different methods on 100-cell datasets simulated using the simulation routine of cnp2cnp with increasing numbers of bins (rows; (A, B, C): 100, (D, E, F): 500, (G, H, I): 1,000, and (J, K, L): 2,000 bins) and at three different noise levels (columns; error rates 0, 0.1, and 0.25). As the number of bins increases, for most methods, the overall performance of all methods steadily increases. The relative performance of methods remains unchanged, with either DICE-bar or DICE-star generally matching or outperforming the other methods depending on the level of noise in the dataset. All results are averaged over 20 datasets.

We also used the cnp2cnp simulator to generate more realistic datasets with additional bins. Unlike the other two simulators, cnp2cnp uses genomes consisting of a single haploid chromosome, though there is a parameter controlling the number of bins (default 100). As Fig S15 shows, the performance of all methods improves as the number of bins increases, with mean performances approaching those observed on the CNAsim datasets. Interestingly, at the two highest bin settings (1,000 and 2,000) and the lowest noise levels (0, 0.1), Lazac becomes the best performing method, very slightly outperforming DICE-bar. Otherwise, the relative performance of the methods remains mostly unchanged, with either DICE-bar or DICE-star outperforming the other methods depending on the level of noise in the dataset. We also find that, at the highest bin setting of 2,000, the method of Minussi et al (2021) matches the performance of DICE-star at all noise levels. This is likely an artifact of the mutation model of the cnp2cnp simulator being limited in how copy numbers change in individual events.

### DICE-bar and DICE-star are highly scalable

**Figure 5.**
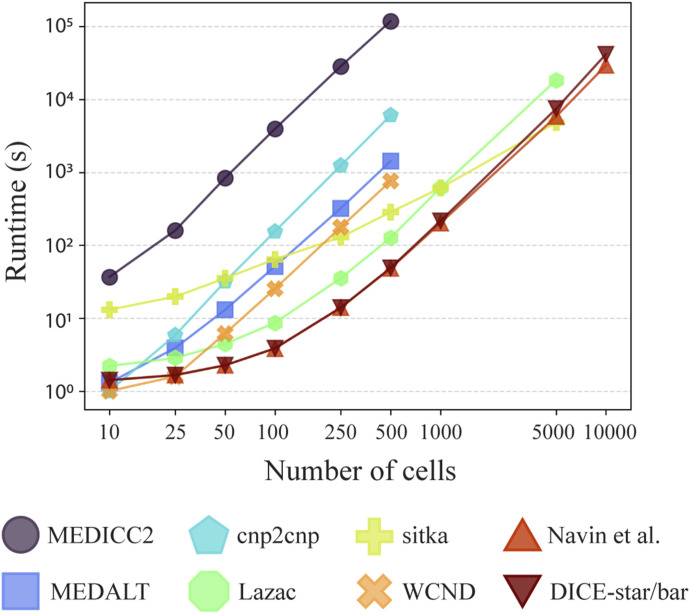
Running time and scalability. Running times are shown for MEDICC2, MEDALT, cnp2cnp, Lazac, sitka, WCND, Navin et al, Minussi et al, DICE-bar, and DICE-star on noise-free datasets with varying numbers of cells. Running times for DICE-bar, DICE-star, and the method of Minussi et al (2021) are identical and are shown together. The x-axis is the log-scaled number of cells in the dataset, and the y-axis is the log-scaled runtime in seconds. Observe that DICE-star and DICE-bar are orders of magnitude faster and more scalable than the best MED-based methods. All reported times are averaged over 20 runs executed using a single core of an Intel Xeon 2.1 GHz processor.

**Figure S16.**
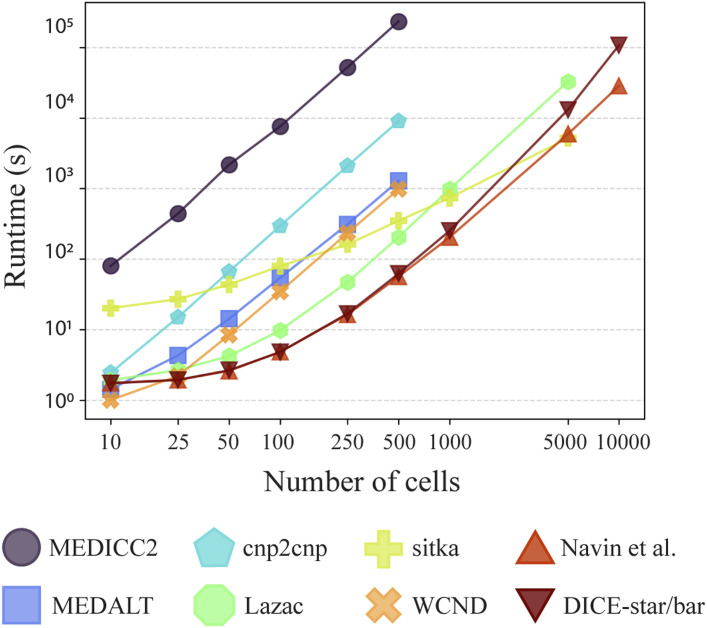
Running time and scalability on noisy data. Running times are shown for MEDICC2, MEDALT, cnp2cnp, Lazac, sitka, WCND, Navin et al, Minussi et al, DICE-bar, and DICE-star on high-noise datasets with varying numbers of cells. Running times for DICE-bar, DICE-star, and the method of Minussi et al (2021) are identical and are shown together. The x-axis is the log-scaled number of cells in the dataset, and the y-axis is the log-scaled runtime in seconds. All reported times are averaged over 20 runs executed using a single core of an Intel Xeon 2.1 GHz processor.

In addition to their accuracy, DICE-bar and DICE-star are also highly scalable and computationally efficient, handling thousands of cells in a matter of minutes. Fig 5 reports running times of the different methods on noise-free datasets with varying numbers of cells (and generated using default values for other parameters). All methods were run on a single core of an Intel Xeon 2.1 GHz processor with 64 GB of RAM. As the figure shows, both DICE-bar and DICE-star, are much faster and more scalable than all MED-based methods. For example, DICE-bar and DICE-star are both over 1,000 times faster than MEDICC2 on the 500-cell datasets. The methods of Navin et al and Minussi et al, being similarly distance-based, have nearly identical running times as DICE-bar and DICE-star. Observe that sitka becomes the fastest method for 5,000 cells; however, this result is misleading because the running time of sitka depends on the number of steps used in its Markov chain Monte Carlo search heuristic, which was kept constant across all runs. We also assessed the impact of CNA estimation error (i.e., noise) on running times and found that all methods report a slight increase in running time (Fig S16).

### Application to large-scale scDNA-seq cancer datasets

Given the robustness of DICE-star to error-prone data, we applied the method to 35 previously published whole-genome scDNA-seq datasets from human triple-negative breast cancer or high-grade serous ovarian cancer samples (Funnell et al, 2022). The median sequencing coverage was ≈. Th per cell, and the number of cells per dataset varied between a minimum of 65 and a maximum of 6,033 with a mean and median of 1,232 and 631, respectively. As we describe below, our analysis demonstrates that DICE-star produces cell lineages trees with greater concordance to previously reported clonal structures, and with a higher degree of similarity between the CNPs of closely related cells, compared with existing approaches.

To study copy number evolution, the original study generated allele-specific CNPs with 500 kbp bins using the SIGNALS method. Cells were then clustered by their CNPs to identify the major clonal populations of each tumor, and copy number evolution was studied by constructing phylogenetic trees using sitka (Salehi et al, 2023). In a later study, cell lineage trees constructed using Lazac on the same CNPs were shown to be more concordant with the originally identified clonal populations (Schmidt et al, 2023).

Using the same CNPs, we used DICE-star to generate cell lineage trees for all 35 tumors. This required 24.7 h using a single core of an Intel Xeon 2.1 GHz processor. Lazac required 166.5 h to compute the trees, and we obtained the sitka trees directly from Funnell et al (2022). We find that the trees inferred by each method on the same dataset differ considerably from one another, with mean NRFDs of 0.95, 0.984, and 0.984 for DICE-star w/Lazac, DICE-star w/sitka, and Lazac w/sitka, respectively. However, because the NRFDs may be skewed by the considerable number of cells in these datasets, we also compute normalized Quartet distances between each pair. This results in mean values of 0.421, 0.524, and 0.512 between the respective pairs, indicating that the DICE-star and Lazac phylogenies are the most similar. Following Schmidt et al (2023), we conduct two distinct evaluations to quantitatively evaluate the cell lineage tree computed by DICE-star, Lazac, and sitka.

**Figure 6.**
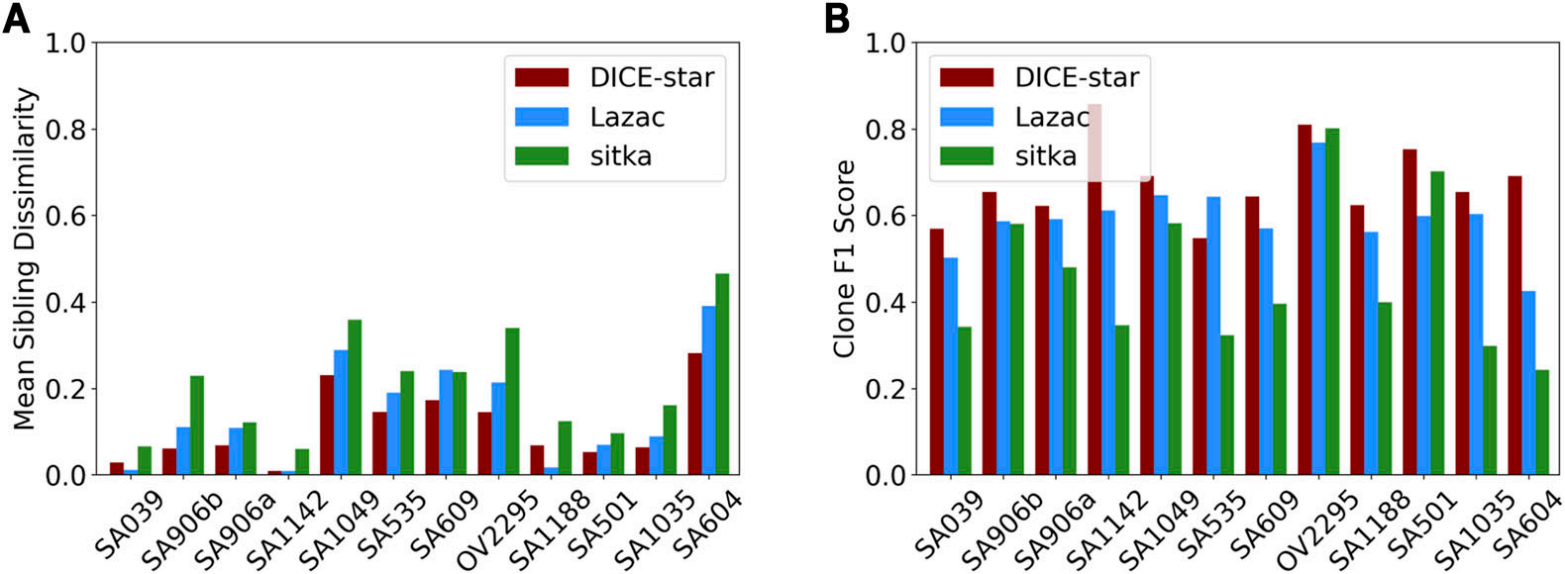
Summary statistics of inferred cell lineage trees on large single-cell datasets. Summary statistics are shown for trees inferred by DICE-star, Lazac, and Sitka on the 12 single-cell datasets of breast and ovarian tumors from Funnell et al (2022) with more than 1,000 cells. **(A)** Mean sibling dissimilarity, defined as the mean normalized hamming distance between siblings in the inferred trees. **(B)** Clone F1 score, defined as the maximal F1 score between clades in the inferred trees and existing clone assignments of Funnell et al (2022).

First, we use the sibling dissimilarity metric of Schmidt et al (2023) to evaluate the cell lineage trees. This metric, defined as the mean normalized Hamming distance between pairs of siblings in the respective phylogenies, was used by Schmidt et al (2023) to show that the increased resolution of Lazac trees (which, like DICE-star trees, are fully resolved) over sitka trees (which are often unresolved/multifurcated) was meaningful. Using the same metric, we find that DICE-star has lower (better) sibling dissimilarity than Lazac and sitka in 31/35 datasets, with means of 0.155, 0.2, and 0.282 for DICE-star, Lazac, and sitka, respectively (Fig 6A). This highlights the higher degree of similarity between the CNPs of closely related cells in the DICE-star trees compared with Lazac and sitka trees.

And second, we investigate the degree of concordance between the computed cell lineage trees and the clonal populations reported in the original publication (Funnell et al, 2022). As with the simulation study, we compute the maximal F1 scores between clades in the trees and cell assignments given by the clones. We find that the DICE-star trees show greater F1 score than those of Lazac and sitka for 24/35 tumors, with means of 0.73, 0.656, and 0.609 for DICE-star, Lazac, and sitka, respectively, across the 35 datasets (Fig 6B). Thus, the phylogenies produced by DICE-star are more congruent with previously identified clone assignments compared with the other methods.

### Application to two breast cancer datasets

We further apply DICE-star to two previously published scDNA-seq datasets of triple-negative breast cancer patients (Navin et al, 2011), referred to as T10 and T16, and perform a qualitative assessment of the resulting cell lineage trees. The mean coverage for T10 and T16 was ≈0.08 and ≈0.13, respectively, and raw sequencing data were obtained for both patients using the SRA toolkit (Leinonen et al, 2011) in the form of fastq files for each cell. Following standard practices, sequencing reads were aligned to the hg38 human reference genome using BWA (Li, 2013 *Preprint*) and filtered for quality using Samtools (Li et al, 2009).

In the previous study of Navin et al (2011), FACS was used to study the distribution of ploidy across the single-cell populations, revealing a majority diploid fraction and smaller aneuploid fractions for both tumors, and a hypodiploid fraction in T10. Using this information, Navin et al (2011) selected 100 flow-sorted single cells each from T10 and T16, taking care to include representatives from the various ploidy fractions and anatomical sectors. For T16, this included 48 cells from a paired metastatic liver carcinoma, while all 100 cells from T10 were sampled from the primary site. To investigate population structure, Navin et al (2011) constructed evolutionary trees using Neighbor Joining based on pairwise Euclidean distances between CNPs derived in situ (referred to as the method of Navin et al throughout this work), revealing large well-defined clades in both tumors. In particular, the subpopulations induced by the four major clades of their T10 tree map perfectly to the represented ploidy fractions, namely a diploid, hypodiploid, and two aneuploid populations. Similarly, in their T16 tree, two major clades clearly separate the diploid and aneuploid populations, and the aneuploid clade is further subdivided into two smaller subpopulations, representing cells from the primary and metastatic sites.

**Figure 7.**
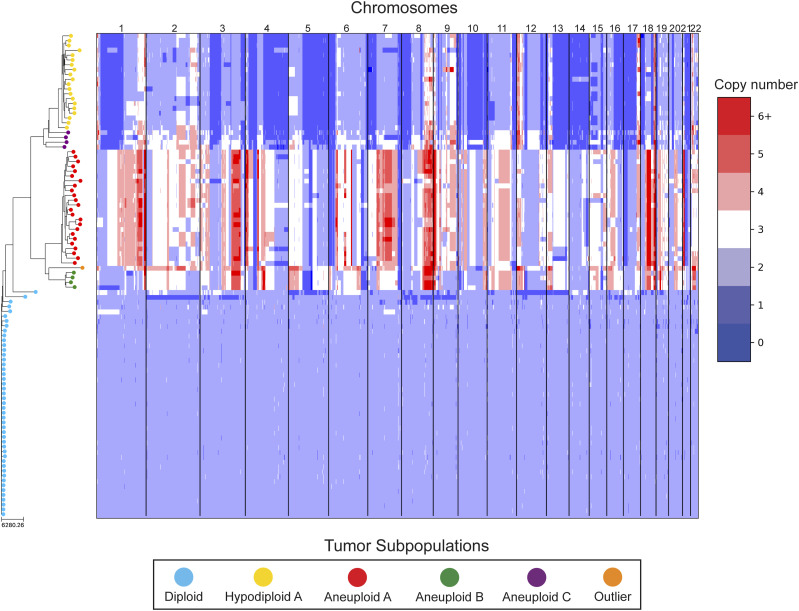
DICE-star tree of T10 aligned to a heatmap of whole-genome single-cell CNPs. Leaf nodes are shaded to match their corresponding clones derived by applying k-means clustering to the CNPs. The main clonal populations shown are defined by their ploidy: diploid (blue), hypodiploid (yellow), aneuploid A (red), and aneuploid B (green). Further increasing the number of clusters reveals an additional aneuploid clonal population (purple) from which the main hypodiploid population diverged, and a high-ploidy outlier cell (orange) placed in between aneuploid populations A and B.

**Figure S17.**
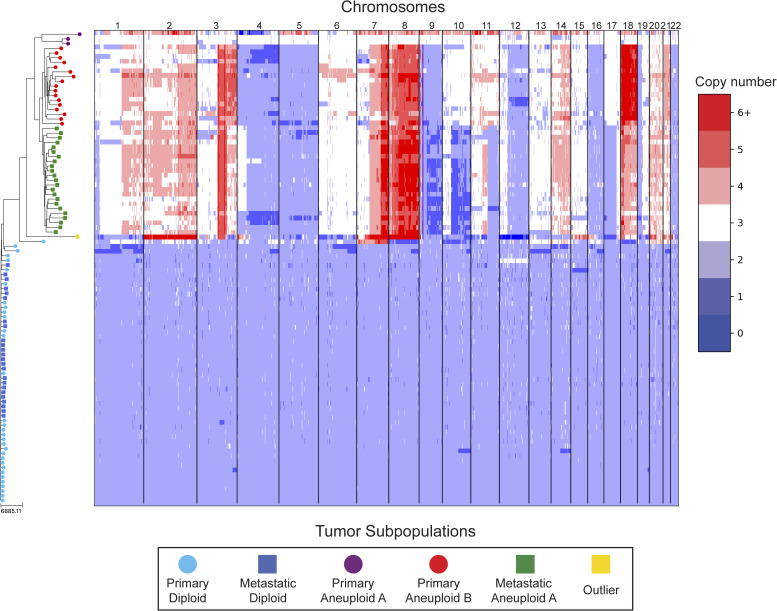
DICE-star tree of T16 aligned to a heatmap of whole-genome copy number profiles. Leaf nodes are shaded to match their corresponding clones derived from applying k-means clustering to the copy number profiles. The main clonal populations displayed are defined by their ploidy: diploid (blue), primary aneuploid B (red), and metastatic aneuploid (green). Further increasing the number of clusters reveals an additional small aneuploid population from the primary site (purple) that is significantly well-separated from the other clones, and an outlier cell sampled from the metastatic site whose copy number profiles contain a multitude of unique chromosomal CNAs (yellow). Both appear to have diverged from the previously reported populations far earlier than with each other, and may represent undersampled rare populations.

**Figure S18.**
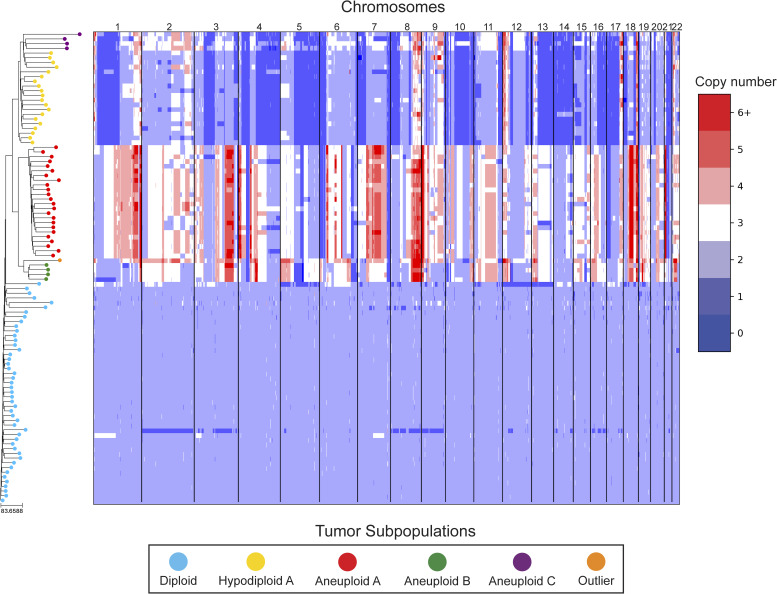
MEDICC2 tree of T10 aligned to a heatmap of whole-genome single-cell CNPs. Similar to Fig 7, leaf nodes are shaded to match their corresponding clones derived by applying k-means clustering to the CNPs.

**Figure S19.**
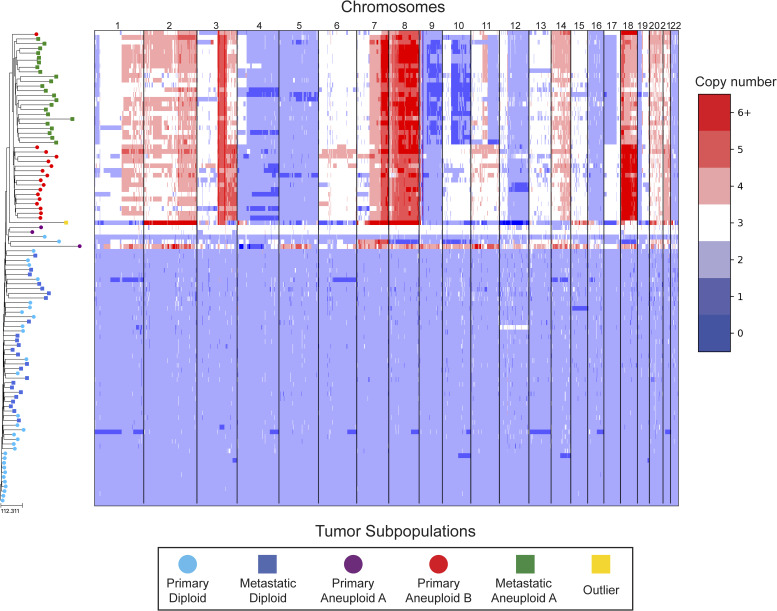
MEDICC2 tree of T16 aligned to a heatmap of whole-genome single-cell CNPs. Similar to Fig 7, leaf nodes are shaded to match their corresponding clones derived by applying k-means clustering to the CNPs.

**Figure S20.**
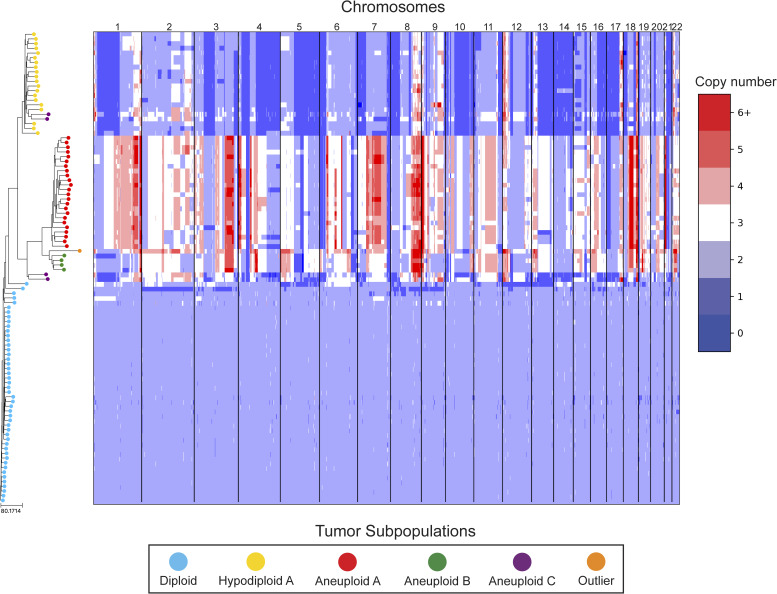
Navin et al tree of T10 aligned to a heatmap of whole-genome single-cell CNPs. Similar to Fig 7, leaf nodes are shaded to match their corresponding clones derived by applying k-means clustering to the CNPs.

**Figure S21.**
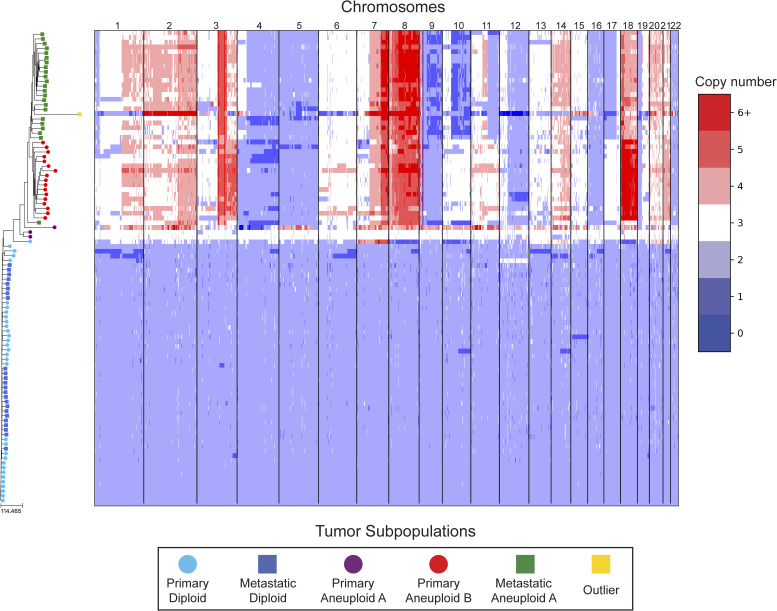
Navin et al tree of T16 aligned to a heatmap of whole-genome single-cell CNPs. Similar to Fig S24, leaf nodes are shaded to match their corresponding clones derived by applying k-means clustering to the CNPs.

In our reanalysis of these datasets, we used SCOPE (Wang et al, 2020) to generate CNPs from the aligned and processed sequencing reads. We initially set the fixed bin size to 100 kbp as we deemed this value to be large enough to overcome most fluctuations given the coverage while remaining close to the median variable bin size used in the original study of 54 kbp. After quality control and normalization, the resulting CNPs totaled 24,779 and 24,534 bins for T10 and T16, respectively. Cell lineage inference using DICE-star took only a few seconds for both tumors on a personal laptop computer, while MEDICC2 took ∼5 h on each dataset. To root the trees, a “dummy” true diploid was included into the population and subsequently set as the outgroup. Figs 7 and S17 show DICE-star trees over 100 kbp bins for T10 and T16, respectively. For comparison, we also generated trees using MEDICC2 and reapplied the method of Navin et al on the SCOPE CNPs (Figs S18, S19, S20, and S21).

On both datasets, we find that all three trees depict a near-identical partitioning of the cells into four and three analogous divergent clades in T10 and T16, respectively, with equivalent macro-evolutionary relationships. When classified by ploidy, the clones induced by the divergent clades align with the known population fractions from the original analysis and are referred to as such. Ignoring variations within the major clades, the differences between the trees pertain to the placement of a small number of contentious cells in each dataset. In T10, both the DICE-star and MEDICC2 trees describe a visually distinct four-cell cluster in close proximity to the hypodiploid clade (labelled aneuploid C in the figures), appearing as ancestral in the former and descendent in the latter. In contrast, these cells cluster poorly in the Navin et al tree. The heatmap (Fig 7) reveals a number of shared large-scale aberrations unique to the four cells, including amplifications on chromosomes 2p, 11, 12, and 18, and may therefore indicate a potential unreported clonal population. In T16, the DICE-star and Navin et al trees describe a three-cell cluster ancestral to the two main divergent clades (labelled primary aneuploid A in the figures), while these cells appear within the normal diploid clade in the MEDICC2 tree. Upon closer inspection, we find that while one of the cells contains numerous unique gains and losses, the other two have a stable triploid state across the genome. While potentially representing poorly sampled populations, the latter two cells are likely artifacts caused by doublets or abnormally high number of reads across the genome.

To further validate these findings, we identified clones independently from the phylogenies by applying k-means clustering to the CNPs of each cell. To determine the optimal number of clusters, we enumerated over all values 3,..., 10 and found that using the number of clusters k = 6 and k = 5 resulted in the highest Silhouette Coefficient (Rousseeuw, 1987) for T10 and T16, respectively. In each dataset, the contentious set of cells is grouped together within a distinct cluster, with the remaining clusters corresponding to the consensus major divergent clades and a single outlier cluster. To quantify concordance with the trees, we computed the adjusted rand index (ARI) between the k-means clusters and clusters induced by clades in the tree (see the Materials and Methods section). In T10, the ARI values were 1.0, 0.991, and 0.937 for DICE-star, MEDICC2, and Navin et al, respectively, and for T16 the values were 1.0, 0.945, and 1.0. Overall, the k-means clusters substantiate both the separation of the contentious sets of cells from the other populations and their close-relatedness in the trees; however, this consistency may also reflect artifacts in the CNPs.

**Figure S22.**
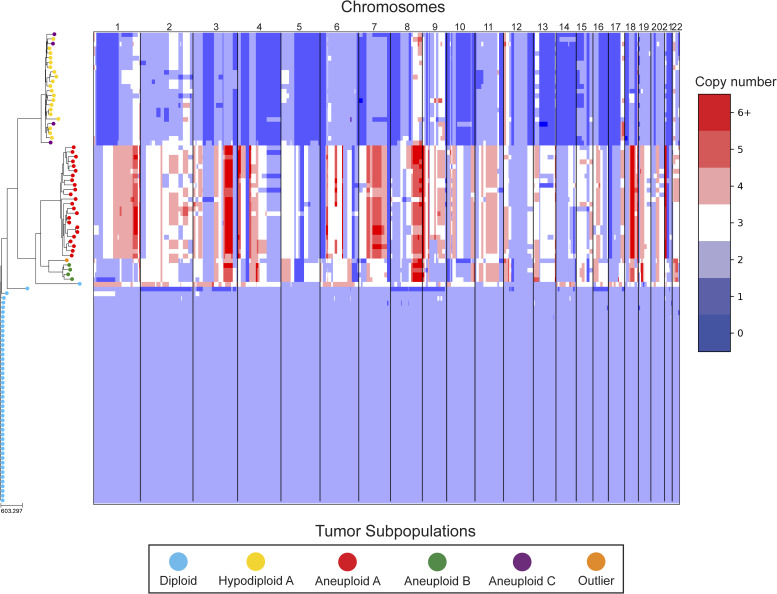
DICE-star tree of T10 w/1 Mbp bin sizes aligned to a heatmap of whole-genome single-cell CNPs. For comparison, leaf nodes are shaded to match the clones derived using k-means clustering on the 100 kbp bin CNPs. Note that the four-cell cluster identified in the 100 kbp CNPs (Aneuploid C) is no longer distinct when using the 1 Mbp CNPs.

**Figure S23.**
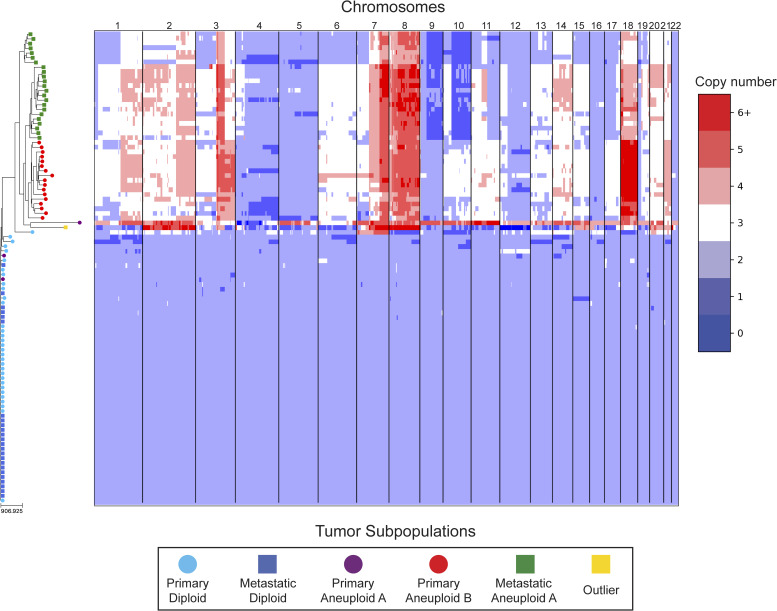
DICE-star tree of T16 w/1 Mbp bin sizes aligned to a heatmap of whole-genome single-cell CNPs. For comparison, leaf nodes are shaded to match the clones derived using k-means clustering on the 100 kbp bin CNPs. Note that the three-cell cluster identified in the 100 kbp CNPs (Primary Aneuploid A) is no longer distinct when using the 1 Mbp CNPs. Also, observe the formation of an entirely new clade, composed of cells belonging to the Metastatic Aneuploid A population in the 100 kbp tree.

As a final qualitative assessment, we investigate the impact of bin size by exploring changes in the DICE-star trees generated with CNPs with larger bins. The difference in read count fluctuations due to bin size means that SCOPE, which estimates ploidy statistically, may produce CNPs which differ substantially for separate sizes. While consistency in the placement of cells across bin size likely indicates reliability, differences are also useful in identifying potentially problematic cells or regions. In addition to 100 kbp, we generated CNPs with SCOPE using bin sizes 250, 500 kbp, and 1 Mbp. The DICE-star trees and heatmaps over the 1 Mbp CNPs are shown in Figs S22 and S23 (250 and 500 bkp CNPs not shown). In T10, we find that the four main clades remain consistent across bin sizes. However, the contentious four-cell cluster becomes less distinctive as bin size increases, and becomes fully integrated into the hypodiploid clade by 1 Mbp. This is reflected in the 1 Mbp CNPs, which no longer show the same unique large-scale aberrations as those in the 100 kbp CNPs. In T16, we similarly find consistency in the three main clades across bin sizes but no longer observe the contentious three-cell cluster, which using 1 Mbp correspond to one outlier and two diploid cells. Interestingly, for >100 kbp bins, we observe a new well-separated clade composed of six cells previously found in the metastatic aneuploid clade. This new clade is ancestral to the existing aneuploid cells and uniquely lacks the large-scale aberrations on chromosomes 1p, 3p, 6, and 15. These findings highlight the importance of considering the effect of bin size on downstream interpretation of cell lineage trees.

Overall, this analysis further demonstrates how DICE-star can reconstruct high-quality cell lineage trees for real datasets and can do so thousands of times faster than existing gold-standard MED-based methods like MEDICC2.

## Discussion

In this work, we introduced two new methods, DICE-bar and DICE-star, for reconstructing tumor cell lineage trees from single-cell copy-number data. Both methods are based on novel, easy-to-compute distance measures, and outperform the current state-of-the-art in terms of accuracy and scalability. Using an extensive simulation study, we showed that DICE-bar matches or improves upon the accuracies of existing methods across nearly all experimental conditions on noise-free data, and that DICE-star substantially improves upon all methods, including DICE-bar, on nearly all datasets with noise/error levels similar to those observed in inferred CNPs on real sequence data. Remarkably, we also found that DICE-bar matches or exceeds the accuracies of MED-based methods across nearly all conditions and noise levels. Importantly, DICE-bar and DICE-star are also much faster and more scalable than MED-based methods.

The results of our simulation study also demonstrate the drastic effect that noise in CNPs has on the ability of MED-based methods, based on nuanced models of copy number evolution, to effectively reconstruct the underlying phylogeny. At the same time, we find that DICE-star remains highly tolerant to noise/error in the input CNPs, and outperforms those other methods for both low and high levels of noise. The lack of robustness to noise observed in MED-based methods is not entirely unexpected. Current limitations in scDNA-seq coverage necessitate the use of large bins to overcome poor resolution, and this can lead to single-cell CNPs that differ by only a small number of events. Consequently, even low levels of noise can disproportionately affect MED distance, particularly between cells that are closely related. This may explain why some MED-based methods, notably MEDICC2, appear to be more robust to noise when considering clone detection but show high sensitivity to noise when considering tree reconstruction accuracy. Still, the fact that DICE-bar, which is based on breakpoints and therefore similarly affected by noisy CNPs, generally matches or outperforms existing MED-based methods on both noise-free and noisy datasets is surprising.

One of the most important findings of this work is that relatively simple distance-based methods, such as DICE-star or the method of Minussi et al (2021), can produce more accurate tumor cell lineage trees on real scDNA-seq datasets than even the best existing MED-based or breakpoint-based methods. Our analysis of real scDNA-seq cancer datasets highlights the utility of DICE-star in practice, where it was shown to produce results of comparable or greater reliability than those of existing methods. Overall, our findings clearly identify DICE-star, given its robustness to noise, as the method of choice for analyzing real datasets.

Our results suggest several important directions for future research on this problem. We found that the levels of noise typically observed in inferred CNPs on real single-cell datasets can greatly decrease the accuracy of all tumor cell lineage inference methods. Our analysis suggests that DICE-star works very well overall on datasets across a wide range of noise levels. However, DICE-bar can deliver significant improvements over DICE-star when the data is relatively noise free. It may be possible to combine the distance functions of DICE-star and DICE-bar to design a new method that combines the strengths of both approaches. Likewise, it may be possible to adjust how breakpoints are computed to make DICE-bar more robust to noise. We also found that the MED-based method MEDICC2 performs very well when there are only a small number of bins or when the mutation rate is very low, as long as the data is noise free. It would thus be valuable to develop improved MED-based frameworks that are robust to noise, as more robust MED-based methods should, in principle, be able to exceed the accuracies of much simpler methods like DICE-bar and DICE-star. We also note that the use of explicit evolutionary models (such as the MED framework) offers inherent advantages over methods such as DICE-bar or DICE-star in that they enable not only cell lineage tree inference but also detection and placement of individual events along the cell lineage tree. For example, under the MED framework, MEDICC2 can perform ancestral reconstruction, pseudo-ordering of CNAs along edges of the phylogeny, and direct detection of WGDs. Another promising direction is to apply a denoising and/or filtering step to the CNPs before computing distances, which was shown by sitka to be effective at handling noise under certain conditions.

Despite the accuracy and scalability of DICE-bar and DICE-star, their accuracy on very large datasets with thousands of cells may be limited by the local search heuristic used currently for reconstructing the final tree (balME implementation provided in FastME [Lefort et al, 2015]). The tree search may get stuck in local optima, limiting the accuracy of the methods. Improved tree search algorithms under minimum evolution, and strategies for escaping local optima, could therefore improve the accuracy of these methods on large datasets. Finally, some recent approaches have combined CNAs and SNVs under a single model (Satas et al, 2020; Chen et al, 2022; Sollier et al, 2023; Zhang et al, 2023). SNVs, while more challenging to infer accurately from low-coverage single-cell sequencing data than CNAs (Rozhonova et al, 2022), could provide additional information and lead to more accurate tumor cell lineage trees.

## Materials and Methods

### Basic definitions

We assume a reference genome consists of K chromosomes where each chromosome k is partitioned into *nk* ordered bins labelled *1,...,nk*. Here, each bin label corresponds to a unique contiguous subsequence from the reference. Any sampled genomes are aligned to the reference such that all genomes have the same number and sizes of bins for each chromosome. We refer to the number of times a bin appears in a sampled genome as that bin’s copy number, the values of which can be estimated across all samples and bins using methods for detecting CNAs from scDNA-seq data (Mallory et al, 2020a; Wang et al, 2020). The *CNP* for the k th chromosome is a vector $C^{k}=\left(c_{1},...,c_{n_{k}}\right)$ of non-negative integers, where ci denotes the copy number of bin *i* from that chromosome. A whole genome *s* can be described by a set of CNPs $C_{s}=\left\{C_{s}^{1},C_{s}^{2},...,C_{s}^{K}\right\}$. When considering whole human genomes without allosomes, *K = 22* for total copy numbers and *K = 44* for allele-specific copy numbers.

We define a breakpoint to be the difference between two consecutive copy numbers in a CNP *C*. More specifically, the breakpoint *b*_*i*_ *= c*_*i+1*_*-c*_*i*_, where *1 ≤ i ≤ n-1* and *n* is the number of bins in CNP *C*. A breakpoint profile is a vector $B^{k}=\left(b_{1},...b_{n_{k}-1}\right)$ obtained from a CNP *C*^*k*^ that encodes the breakpoints of all consecutive bins from chromosome *k*.

### Description of DICE-bar and DICE-star

#### DICE-star distance function

Given two genomes *s* and *t* with *K* chromosomes each, DICE-star uses the following distance function:

$$d_{Root}\left(s,t\right)=\sum_{k=1}^{K}\sum_{i=1}^{n_{k}}\sqrt{\left|c_{s,i}^{k}-c_{t,i}^{k}\right|}.$$

Note that this distance function is naive to the particularities of copy number evolution. Nonetheless, standard Euclidean and Manhattan distances between CNPs have been used previously for tree reconstruction of real tumor samples by Navin et al (2011) and Minussi et al (2021), respectively. Our novel “Root” distance function above essentially applies the square root to each term of the Manhattan distance. This root distance function is motivated by the following insight: CNAs can amplify a region by multiple copies in a single event, and thus larger changes in copy number do not necessarily imply greater evolutionary distances. Under the standard Manhattan distance, large changes are weighted equivalently to a number of events equal to its magnitude, potentially resulting in misleading evolutionary scenarios. At the same time, it is believed that the probability of a copy number amplification occurring scales inversely with its magnitude (Liu et al, 2009; Lauer et al, 2018). Accordingly, the Root distance attempts to better balance the effect of many low-magnitude CNA events versus few high-magnitude CNA events.

#### DICE-bar distance function

CNAs induce a strong statistical dependence among adjacent loci and multiple events may overlap. This suggests a number of pitfalls with using CNPs directly. First, the length of a CNA determines how many bins will be altered, essentially acting as a weight. Consider the scenario where two genomes differ by a single long event spanning many bins versus if they differed by many small events. It is more likely that the genomes are more closely related if they differ by a single event versus many; however, if the long event exceeds the combined length of the small events, this will not be reflected in their computed distance. Second, independent events that overlap may be weighted less or nearly canceled out. This could occur if a region experiences a gain followed by a deletion event.

DICE-bar addresses this limitation by considering breakpoints rather than the copy numbers themselves. The use of breakpoints enables a more tailored metric for copy number evolution while still allowing for each site to be treated independently, and thus retaining the efficiency of a naive distance measure. In particular, DICE-bar uses the same novel Root distance function used by DICE-star but applies it to breakpoint profiles instead, as defined below:

$$d_{Root}^{\prime }\left(s,t\right)=\sum_{k=1}^{K}\sum_{i=1}^{n_{k-1}}\sqrt{\left|b_{s,i}^{k}-b_{t,i}^{k}\right|}.$$

We note that the choice of distance function used by DICE-bar and DICE-star was based on a preliminary evaluation of several novel and existing alternatives; the results of that preliminary evaluation, based on a small subset of our simulated datasets, appear below.

#### Reconstructing final tumor cell lineage tree

Once all pairwise distances have been computed using the chosen distance function, the next step is to use a distance-based phylogenetic reconstruction approach to obtain the final cell lineage tree. Both DICE-star and DICE-bar use balanced minimum evolution (Desper & Gascuel, 2002), as implemented in the FastME software package (Lefort et al, 2015) to reconstruct the final cell lineage tree. This choice of using balanced minimum evolution was based on a preliminary assessment, where we evaluated four possible distance-based phylogenetic reconstruction algorithms. Further details on the results of that assessment appear below.

### Alternative distance functions and phylogenetic reconstruction methods

#### Selection of appropriate distance functions

We explored the use of four simple and closely related distance functions: Euclidean (*d*_*Euclidean*_), Manhattan (*d*_*Manhattan*_), Root (*d*_*Root*_), and Log (*d*_*Log*_).

##### Standard distances

When applied to CNPs, we refer to these distances as “standard” distances. Given two genomes *s* and *t* with *K* chromosomes each, these standard distances are defined as follows:

$$\begin{array}{rrrrr} d_{Euclidean}\left(s,t\right) & =\sqrt{\sum_{k=1}^{K}\sum_{i=1}^{n_{k}}{\left(c_{s,i}^{k}-c_{t,i}^{k}\right)}^{2}},d_{Manhattan}\left(s,t\right) & =\sum_{k=1}^{K}\sum_{i=1}^{n_{k}}|c_{s,i}^{k}-c_{t,i}^{k}|,d_{Root}\left(s,t\right) & =\sum_{k=1}^{K}\sum_{i=1}^{n_{k}}\sqrt{\left|c_{s,i}^{k}-c_{t,i}^{k}\right|},d_{\mathrm{Log}}\left(s,t\right) & =\sum_{k=1}^{K}\sum_{i=1}^{n_{k}}\log|c_{s,i}^{k}-c_{t,i}^{k}|. \end{array}$$

##### Breakpoint distances

To address the limitations of standard distances, we proposed new distances functions that consider breakpoints rather than the copy numbers themselves. These breakpoint distances are defined analogously to their standard distance counterparts and their formal definitions appear below.

$$\begin{array}{rrrrr} d_{Euclidean}^{\prime }\left(s,t\right) & =\sqrt{\sum_{k=1}^{K}\sum_{i=1}^{n_{k}-1}{\left(b_{s,i}^{k}-b_{t,i}^{k}\right)}^{2}},d_{Manhattan}^{\prime }\left(s,t\right) & =\sum_{k=1}^{K}\sum_{i=1}^{n_{k}-1}|b_{s,i}^{k}-b_{t,i}^{k}|,d_{Root}^{\prime }\left(s,t\right) & =\sum_{k=1}^{K}\sum_{i=1}^{n_{k}-1}\sqrt{\left|b_{s,i}^{k}-b_{t,i}^{k}\right|},d_{\mathrm{Log}}^{\prime }\left(s,t\right) & =\sum_{k=1}^{K}\sum_{i=1}^{n_{k}-1}\log|b_{s,i}^{k}-b_{t,i}^{k}|. \end{array}$$

Of the eight distinct distance functions defined above, six are, to the best of our knowledge, novel (proposed and evaluated for the first time in this work). The other two, standard Euclidean and standard Manhattan, have previously been used to analyze real tumor samples (Navin et al, 2011; Minussi et al, 2021). The DICE software package implements all eight of these distance functions, and DICE-star and DICE-bar correspond to standard-Root (*d*_*Root*_), and breakpoint-Root ($d_{Root}^{\prime }$), respectively.

#### Distance-based phylogeny reconstruction

We evaluated four possible distance-based phylogenetic reconstruction algorithms. These are Neighbor Joining (Saitou & Nei, 1987), unweighted Neighbor Joining (Gascuel, 1997), balanced Minimum Evolution (Desper & Gascuel, 2002), and ordinary least-squares Minimum Evolution (Rzhetsky & Nei, 1993), and are referred to as NJ, uNJ, balME, and olsME, respectively. DICE uses the FastME software (Lefort et al, 2015), which implements all four of these methods, to compute the final cell lineage tree. FastME uses a local search heuristic for balME and olsME; an initial tree is built using an additive taxon procedure, and then an SPR tree search is used to find a topology minimizing the balME or olsME criteria. DICE-bar and DICE-star both use balME to reconstruct the final cell lineage tree.

#### Evaluation of all 32 DICE variants

We evaluated the 32 DICE variants (eight distance functions times four phylogeny reconstruction methods) using a subset of our simulated datasets. Specifically, we used our baseline datasets, simulated using CNAsim with the default parameter values and three different noise levels (no noise, low noise, and high noise) to assess the 32 DICE variants. We used these initial results to identify the best standard-distance method (DICE-star) and the best breakpoint-distance method (DICE-bar) for more thorough evaluation as described in the Results section.

**Figure S24.**
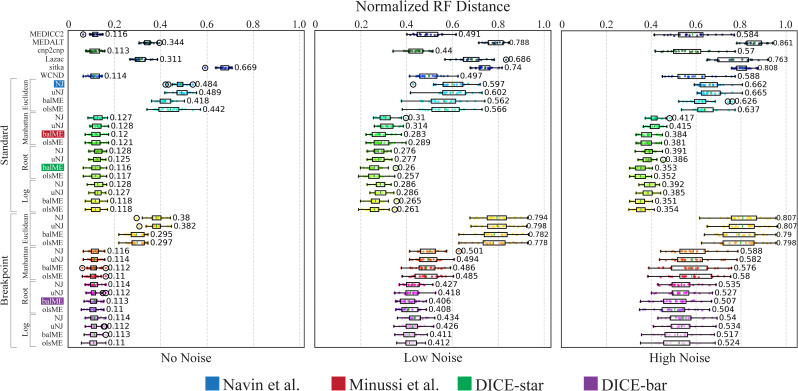
Reconstruction accuracies of all 32 DICE variants and existing methods. Cell lineage tree reconstruction accuracies are shown for all 32 DICE variants and existing methods on simulated datasets with 250 cells and varying levels of noise. The datasets correspond to those appearing in Fig 2 of the main text. Results for datasets with no noise, low noise, and high noise appear in the left, middle, and right panels, respectively. The methods of Navin et al and Minussi et al, which correspond to two of the DICE variants, are highlighted in blue and red, respectively. All results are averaged over 20 simulated datasets.

Fig S24 shows the results of this analysis. These results reveal interesting insights into the relative accuracies of the different DICE variants. First, we find that all Euclidean DICE variants, both standard and breakpoint, are among the worst performing methods at all noise levels. This includes the method of Navin et al (2011), with corresponds to the standard-Euclidean-NJ variant of DICE. Second, we find that all non-Euclidean standard DICE variants show far greater accuracy than all other methods (including all breakpoint DICE variants) on noisy datasets. In contrast, on noise-free data, the non-Euclidean breakpoint DICE variants generally match or outperform all other methods. Third, among DICE variants, we find that the root and log distances show a slight advantage in performance over Manhattan distance, which includes the method of Minussi et al (2021), and that balME- and olsME-based variants perform better than the NJ- or uNJ-based ones across all noise levels. Based on these results, we selected the standard-root-balME variant of DICE as the best standard method DICE-star and the breakpoint-root-balME variant of DICE as the best breakpoint method DICE-bar.

We note that some other DICE variants, such as breakpoint-log-balME and standard-root-olsME, show nearly identical performance as DICE-bar and DICE-star, respectively. Among these equally strong variants, we chose root-balME as the basis for DICE-bar and DICE-star because the root distance function is slightly more interpretable than the log distance function, and since balanced minimum evolution (balME) has been observed to perform favorably compared with ordinary least-squares minimum evolution (olsME) in previous phylogenetic studies (Desper & Gascuel, 2002, 2004).

### Description of datasets

In the following, we first describe the extensive datasets simulated using CNAsim, then describe datasets simulated using the two older simulators, and finally describe the real cancer datasets used in this work.

#### Datasets simulated using CNAsim

The most of our datasets were simulated using the recently developed simulator CNAsim (Weiner & Bansal, 2023a), which simulates single-cell genomes along a ground truth cell lineage tree and can directly generate both noise-free and noisy CNPs. Next, we briefly describe the five key steps in the CNAsim simulation framework, discuss key simulation parameters, and justify our default values for these parameters.

##### Generation of ground truth cell lineage tree

Following standard practice, a ground truth cell lineage tree is simulated from an exponentially growing population under neutral coalescence (Hudson, 2002; Beerenwinkel et al, 2007; Bozic et al 2016; Williams et al, 2016; Niida et al, 2020; Posada, 2020), the leaves of which correspond to observed cells in the experiment.

##### Evolution of genome along cell lineage tree

An initial diploid genome is placed at the root of the tree and is represented by an ordered array of uniform-sized regions of *M* base-pairs each, where *M* is the minimum size of a CNA. Previous reports have classified variants as CNAs if its length exceeds 1,000 bp (Feuk et al, 2006; Pollex & Hegele, 2007; Shlien & Malkin, 2009; Upadhyay et al, 2017), which we use as a fixed value for *M*. The default number of chromosomes and chromosome lengths is derived from the GrHg38 human reference genome; however, other custom genome sizes are considered. We add a number of segmental CNAs to the root genome which represents a set of initial mutations that initiate cancer growth (by default, 10× that of normal edges; see below) (Sottoriva et al, 2015; Gao et al, 2016; Saito et al, 2018). Here, segmental CNAs are defined as those smaller than a chromosome arm. In addition, a WGD and/or chromosomal-CNAs can be introduced into the genome of the root cell (tumor founder) that is pervasive across the entire cell population (Bielski et al, 2018). Note that the only cell which can undergo WGD is the tumor founder cell. The genome is evolved along the tree topology, where each node inherits the genome of the parent node in addition to being altered by a Poisson-distributed number of segmental CNAs. Existing estimates place the number of CNAs in human cancers to be in the many tens to hundreds (Gao et al, 2016; Velazquez-Villarreal et al, 2020; Minussi et al, 2021) for a single sample. Accordingly, we use a default of *λ = 2* events per edge as this results in tens of segmental CNAs globally for small trees and hundreds for large trees. Other values for this parameter are explored. Importantly, the total burden of events falls within ranges reported in pan-cancer studies (Harbers et al, 2021). A wide range of possible segmental CNAs is generated by stochastically selecting for each event property. First, the paternal or maternal allele is selected with a draw from a Binomial distribution (default *α = 0.5*). The chromosome is selected at random with probability proportional to chromosome length. Each segmental CNA is chosen to be either a copy number gain or deletion according to a draw from a Binomial distribution. Amplifications and deletions have been reported to occur in relatively equal numbers, albeit with high variance (Zack et al, 2013; Harbers et al, 2021). In general, these numbers tend to scale with one another (Beroukhim et al, 2010), and so we fix the probability of an amplification to be *P* = 0.5. The length of the CNA is drawn from an exponential distribution, as multiple studies have found the frequency of a CNA occurring scales inversely with its length (Itsara et al, 2010; Knouse et al, 2017). We fix the mean length of a CNA to be *β = 5* Mbp based on existing estimates (Beroukhim et al, 2010; Gao et al, 2017; Harbers et al, 2021) while also allowing for meaningful evaluation. After a length is chosen, the starting location is selected on the chromosome uniformly among all possible locations. Lastly, if the event is a copy number gain, the number of additional copies is chosen with a draw from a geometric distribution (Mallory et al, 2020a). The mean number of additional copies is fixed to be *δ = 2* reflecting previous observations (Liu et al, 2009; Lauer et al, 2018), and all additional copies are inserted in tandem with the original.

##### Larger-scale CNAs and inclusion of clonal structures

Beyond segmental CNAs, the genomic landscape in tumors can be driven by large-scale CNAs occurring at the chromosome-arm or whole-chromosomal level. Chromosomal CNAs are known to be an influential factor in the expansion of diverging lineages and have previously been used as a criteria to define distinct clonal populations (Velazquez-Villarreal et al, 2020). Following this definition, clonal expansions can be introduced into the population by selecting lineages which undergo a unique set of chromosomal CNAs. In particular, ancestral nodes are selected from the tree and, in addition to segmental CNAs, are subjected to both whole-chromosomal and chromosome-arm CNAs, thereby significantly separating their descending lineage from the rest of the tree (Secrier et al, 2016; Zaccaria & Raphael, 2021). To achieve this, a fixed number of ancestral nodes are chosen based on the size of the clade. Note that cells within clonal lineages do not have identical genomes as they continue to accumulate segmental CNAs. To simulate chromosomal CNAs, we first determine if the event affects a chromosome arm or a whole chromosome with a Binomial distribution. We fix *P* = 0.75 with probability weighted in favor of chromosome-arm CNAs, as these are thought to be more common (Taylor et al, 2018). The specific chromosome or chromosome-arm is selected uniformly at random. Both chromosome-arm CNAs and whole-chromosomal CNAs can either be a deletion or duplication event, but not an amplification (Beroukhim et al, 2010), and is determined by a draw from a Binomial. By default, we set *P* = 0.5 for an even distribution of duplications and deletions. However, in the presence of WGD, we set *P* = 0.8 in favor of deletions, as the median ploidy of tumors having undergone WGD is close to 3 (Bielski et al, 2018). We note that clonal structures (or chromosomal CNAs) are not included in our default simulation parameter settings; instead we simulate additional datasets to explore the impact of varying numbers of clones.

##### Generation of noise-free CNPs

Upon completing the tree traversal, CNPs are generated for each leaf node by “mapping” regions back to the starting diploid genome and grouping them into fixed-size bins. On real low coverage data, bin sizes typically are in the order of 500 kbp (Garvin et al, 2015) to 5 Mbp (Zaccaria & Raphael, 2021). Accordingly, the default bin size is set to 1 Mbp, but we also evaluate other bin sizes.

##### Generation of noisy CNPs

In the last step, noise/error is introduced into the CNPs. This is important because copy numbers estimated from real sequencing data can be highly error-prone (Mallory et al, 2020a). The simulator models two primary sources of noise in real CNPs. First, the “boundary” model adds poor resolution at the edges of contiguous segments with the same copy number, which has previously been reported to be the main sources of error in copy number detection algorithms (Garvin et al, 2015; Mallory et al, 2020a). Second, the “jitter” model adds random fluctuations due to biases in sequencing technologies which often affect downstream analysis, for example, uneven coverage (Navin, 2014). A more complete description of the noise models appears later in this section.

#### Selection of appropriate noise parameters

The benchmarking study of Mallory et al (2020a) evaluated the breakpoint detection accuracy of several existing CNA detection methods across a multitude of sequencing technologies and experimental conditions, and reported precision and recall values in the ranges of 0.4–0.75 and 0.5–0.8, respectively, for the best performing method. Based on this benchmarking study, we use two different noise levels, low and high, in our simulation. For the *low noise* setting, we used a boundary error rate, *r*_*b*_ of 0.02 and a jitter error rate *r*_*j*_ of 0.1, which results in a precision and recall of 0.710 and 0.761, respectively. For the *high noise* setting, we used *r*_*b*_ *= 0.04* and *r*_*j*_ *= 0.1*, which results in precision and recall of 0.652 and 0.668, respectively. We note that while these parameter settings result in a significant loss in breakpoint detection accuracy, the vast most of copy numbers remain unchanged because the number of breakpoints between contiguous segments is far less than the total number of bins. On average, only 0.064% of bins have an altered copy number in the low noise setting, and only 1.05% of bins have an altered copy number in the high noise setting. Further details on how these boundary and jitter error rates were selected and how precision and recall values were derived for the simulated noisy datasets appear below. In addition, we also generated datasets with varying jitter and boundary error rates to assess their impact on the reconstruction accuracies of different methods.

#### Simulation parameter ranges and defaults

To assess the robustness of the methods on different dataset types, scales, evolutionary conditions, error-rates, etc., we systematically explored the impact of changing key simulation parameter values. For each distinct combination of parameter settings, we generated 20 independent replicates and all reported results are averaged over these 20 replicates. A list of the key simulation parameters whose impact was systematically explored, along with their ranges and default values, appears below.

• Number of cells: *n* ∈ {10, 25, 50, 100, 250, 500, 1,000, 5,000, 10,000}; default 250.
• Number of chromosomes: *x* ∈ {1, 2, 5, 10, 22}; default 22.
• Chromosome length (in Mbp): *y* ∈ {50, 100, 200, 500, 750, 1000}.
  o If *x* = 22, then *y* uses lengths from the human reference genome hg38.
  o If *x* ≠ 22, then the default is *y* = 100 for every chromosome.
• Mean number of CNAs per edge: *λ* ∈ {0.5, 1, 2, 3, 4, 5}; default 2.
• Bin size (in kbp): *b* ∈ {500, 1,000, 2,000, 5,000, 10,000}; default 1,000.
• Number of clones: *c* ∈ {0, 2, 4, 6}; default 0.
• WGD: *w* ∈ {True, False}; default false.
• Boundary error rate: *rb* ∈ {0, 0.01, 0.02, 0.04, 0.06, 0.08, 0.1, 0.15}; default values of 0, 0.02, and 0.04 for “no noise,” “low noise,” and “high noise” datasets, respectively.
• Jitter error rate: *rj* ∈ {0, 0.05, 0.1, 0.15}; default values of 0, 0.1, and 0.1 for “no noise,” “low noise,” and “high noise” datasets, respectively.

#### Noise models and selection of noise parameters

We begin by providing a brief description of the noise models used in CNAsim. After generating the ground truth CNPs, CNAsim introduces noise/error using two models. First, as part of the boundary model, the boundaries of contiguous segments with the same copy number are extended or shrunk such that bins at the edges of segments are converted into their neighboring segment. This is motivated by previous studies which have shown a concentration in copy number error over bins near segment boundaries (Mallory et al, 2020b). For each distinct segment, the new length of the segment is drawn from a Gaussian distribution with mean equal to the segment length, and a SD equal to the segment length multiplied by the error rate. Second, as part of the jitter model, random jitter is introduced to each bin independently. The intention is to mimic random fluctuations in read counts across the genome that are common to scDNA-seq technologies, the primary cause being nonuniform coverage. For each bin with copy number *c*, a new copy number is assigned based on a draw from a Gaussian distribution with mean to equal *c* and SD equal to *c⋅r*, where *r* is the error rate given by the user. Further details on these noise models appear in Weiner and Bansal (2023a).

The error rates used in CNAsim for generating noisy data were selected to reflect the performance of existing CNA detection methods reported in the benchmarking study of Mallory et al (2020a). This study computed the predicted copy number breakpoints of each method from sequencing data generated from simulated genomes where the ground truth copy number breakpoints are known. By comparing the genomic locations of the predicted and ground truth breakpoints, precision and recall values were derived for each of the evaluated methods. Across all experiments, the best performing methods in the study achieved precision and recall values in the ranges of 0.4–0.75 and 0.5–0.8, respectively.

For the selection of appropriate error rates in CNAsim, we chose values for the noise parameters *r*_*b*_ and *r*_*j*_ that result in precision and recall values within these ranges. When running CNAsim with a given combination of noise parameters, we can output the “clean” CNPs in addition to the noisy CNPs. Thus, for a given combination of *r*_*b*_ and *r*_*j*_, we can compute precision and recall values using the clean and noisy CNPs. In particular, given a clean CNP *C=(c*_*1*_*,...,c*_*n*_*)* and noisy CNP $C^{\prime }=\left(c_{1}^{\prime },...,c_{n}^{\prime }\right)$, we apply the following procedure:

1 Compute the breakpoint profiles *B* = (*b*_1_,...,*b*_*n*−1_*)* and $B^{\prime }=\left(b_{1}^{\prime },...,b_{n-1}^{\prime }\right)$.
2 Compute the sets of informative breakpoints *X* = {*i*:*i* ∈ 1...*n*−1 and *b*_*i*_ ≠ 0*}* and $X^{\prime }=\left\{i:i\in 1...n-1\;\text{and}\;b_{i}^{\prime }\neq 0\right\}$.
3 Count the number of true positives (TP), false positives (FP), and false negatives (FN) as |*X*∩*X*′|, |*X*′−*X*|, and |*X*–*X*′|, respectively, and compute precision and recall.

Table S1 details the precision and recall values of all noisy datasets generated with CNAsim. We note that the relatively low precision and recall values for breakpoint detection on noisy CNPs (corresponding to those reported in Mallory et al [2020a]) are not caused by large-scale error in inferred/noisy copy numbers. In fact, the vast most of copy numbers do not fluctuate and are left unchanged in the noisy CNPs. For example, in the boundary error model, because the number of contiguous segments (and therefore also breakpoints) is significantly less than the total number of bins, the boundary model alters a number of bins proportional to the number of segments. Likewise, the jitter error model works by redrawing each bin with copy number *c* from a normal distribution with a mean of *c* and a SD of c*r_*j*_, where *r*_*j*_ is the jitter error rate. This in effect means that the larger (resp. smaller) the copy number, the higher (resp. lower) the chances of observing jitter. For reference, an error rate of *rj = 0.1* will result in 9.55% of bins with copy number 3 to fluctuate, 1.24% of bins with copy number 2 to fluctuate, and a mere 5.73 × 10^−7^% of bins with copy number 1 to fluctuate. On the default high noise setting (jitter error 0.1, boundary error 0.04), on average 1.05% of bins fluctuate compared with the noise-free profiles. On the default low noise setting, this value is 0.064%. These values match existing studies reporting high in-silico raw copy number recall (Funnell et al, 2022).

#### Simulated datasets of existing methods

To rule out any potential bias in the CNAsim datasets, we also created simulated datasets using the simulation frameworks of the two existing methods MEDICC2 (Kaufmann et al, 2022) and cnp2cnp (Cordonnier & Lafond, 2020). Both simulation frameworks take the same general approach as CNAsim; a ground truth tree topology is generated, CNAs are accumulated along each edge, and observed CNPs are derived from the genomes of the leaf nodes. However, there are several important differences between these two previous simulation frameworks and CNAsim related to number of bins, genome resolution, event sizes, event types, error models, etc. A description of key differences appears below.

Using the simulation frameworks of MEDICC2 and cnp2cnp, we generated the same datasets as those outlined in their respective studies (Cordonnier & Lafond, 2020; Kaufmann et al, 2022). We also used these older simulators to create additional datasets with more realistic parameter values for bin size and event rates.

#### Key differences between the three simulation frameworks

CNAsim and the simulation frameworks of MEDICC2 and cnp2cnp all take the same general approach: A ground truth tree topology is generated, CNAs are accumulated along each edge, and observed CNPs are derived from the genomes of the leaf nodes. However, there are several important differences between the two previous simulation frameworks and CNAsim. Some of the key differences include the following.

##### Number of bins

The simulation study of MEDICC2 used at most 10 bins per chromosome with 2 × 22 chromosomes, for a total of 440 bins, and the simulation study of cnp2cnp used at most 250 bins from a single chromosome. In contrast, datasets generated by CNAsim have a more realistic number of bins by default which, at 1 Mbp bin lengths and the 2 × 22 autosome lengths of hg38, comes out to roughly 5,760 bins.

##### Resolution of genome

Both MEDICC2 and cnp2cnp simulation frameworks simulate events over the bins themselves, meaning CNAs always start and end exactly at the boundaries between bins. For comparison, CNAsim simulates events at the region level, which are by default 1,000x smaller than the bins. This results in more realistic CNAs when using CNAsim because real CNAs very rarely start or end at bin boundaries or may be contained within a single bin entirely. We also note that while CNAsim and MEDICC2 simulate genomes with allele-specific copy numbers, cnp2cnp simulates a single haploid chromosome.

##### Magnitudes of copy number gains

Both of the older simulation frameworks model deletions and duplications, but not copy number amplifications of larger magnitudes. CNAsim models the magnitude of a copy number amplification with a geometric distribution such that the higher the magnitude, the lower the probability. This results in duplications still being the most common, but higher magnitude events are possible.

##### Simulation of the cell-lineage tree

Both MEDICC2 and cnp2cnp simulation frameworks construct the ground truth topology by randomly merging cells, compared with the coalescent model used in CNAsim.

##### Zero copy number

While CNAsim allows the deletion of the last copy of a region, cnp2cnp considers various rates of this occurring, and MEDICC2 disallows it completely.

##### Error in CNPs

The simulation framework of cnp2cnp has a simple noise-model similar to the jitter model of CNAsim. The simulation framework of MEDICC2 can only generate noise-free CNPs and does not explore noisy CNPs. CNAsim has the most complex noise model and includes both jitter error and boundary error.

##### Event types

The simulation framework of cnp2cnp only uses a single chromosome and is limited to segmental duplications and deletions. The simulation framework of MEDICC2 models a variety of events including segmental duplications and deletions, WGD, and whole-chromosomal duplications and deletions. In addition, MEDICC2 models several events that are not included in CNAsim, namely balanced and unbalanced translocations, insertions, and inversions.

#### Real datasets

In addition to using simulated datasets, we also evaluate our most robust method, DICE-star, on a total of 37 publicly available real cancer datasets. The first 35 datasets are derived from breast and ovarian tumors and were generated using DLP+ whole-genome scDNA sequencing (Funnell et al, 2022). The latter two are derived from two breast cancer patients, T10 and T16, and were generated using whole-genome amplification-based SNS technology (Navin et al, 2011).

### Quantifying concordance between cell lineage trees and clusters

Given a cell lineage tree and a set of clusters inferred independently from the tree, we quantify the concordance between clades in the tree and the clusters using the ARI. To compute the ARI, we induce a partitioning of the cells based on the topology of the tree as follows.

Given a tree *t* and a set of clusters *C*, for some cluster *c∈C* we identify the subtree *s∈T* which shows maximum F1-score with respect to *c*. The cells in *s* then represent a clade-induced cluster used to compute the ARI. Once *s* is selected, the clade is pruned from the tree and this process continues until no clusters remain or until the tree is empty. As the order in which clusters are considered affects subsequent cluster selections, we enumerate over all possible orderings and choose the ordering which maximizes the ARI.

### Specific commands used for running existing methods

The specific commands and scripts used for running MEDICC2 (Kaufmann et al, 2022), MEDALT (Wang et al, 2021), cnp2cnp (Cordonnier & Lafond, 2020), sitka (Salehi et al, 2023), and Lazac (Schmidt et al, 2023) in our experimental analysis are available in the DICE GitHub repository (https://github.com/samsonweiner/DICE). These methods were all run in accordance to their respective software manuals or readme files using suggested or default options. Note that MEDICC2 also provides options for reconstructing ancestors on the inferred phylogeny. To enable a fair comparison of running times, we enabled the –topology-only flag of MEDICC2 so as to output the tree topology only (skips reconstructing ancestors), and also enabled the –no-plot flag to skip generating visual plots. For WCND (Zeira & Raphael, 2020), there does not exist a fully operable software package or user manual and we therefore describe how we used WCND below.

The WCND code repository on GitHub provides only high-level functions and does not include examples or a guide to defining weight functions. Because of this, we used the “semi_directed_cnd” function to compute unweighted MED distances over all chromosomal CNPs of each pair of cells, set the total distance between a pair of cells as the sum of the chromosomal unweighted MED distances, and reconstructed the tree from the pairwise distances using neighbor joining.

Sitka was run as in Salehi et al (2023), using the first four steps described in the GitHub readme file. For steps 1, 2, and 4 (jitter correction, loci filtering, and point estimation), we run the commands with the parameters exactly as they appear in the readme. Following the approaches of (Kaufmann et al, 2022; Schmidt et al, 2023), for step 3 (tree inference), we use the same parameter values used in the original publication on real datasets (see Table S1 from Salehi et al [2023]).

We note that the simulated datasets generated with CNAsim and the MEDICC2 simulator contain allele-specific CNPs, while the datasets generated with the cnp2cnp simulator only contain total CNPs. Thus, in the case of datasets generated with the cnp2cnp simulator, all methods receive total CNPs as input. For datasets simulated with CNAsim and the MEDICC2 simulator, allele-specific CNPs are provided as input to DICE-bar, DICE-star, Navin et al, Minussi et al, MEDICC2, Lazac, and WCND, which can all explicitly read multiple copy numbers from different alleles at the same genomic position. For cnp2cnp, sitka, and MEDALT, which all expect a single copy number at any one locus, these allele-specific CNPs are summed together and passed to those method as total CNPs.

## Supplementary Material

Reviewer comments

## Data Availability

Raw sequencing data for the 35 breast and ovarian cancer datasets of Funnell et al (2022) are available from the European Genome-Phenome under study ID EGAS00001006343. The corresponding processed data (CNPs and read count matrices) for each dataset was obtained from Williams (2022). Raw sequencing data for the two breast cancer datasets of Navin et al (2011) are available from the NCBI Sequence Read Archive under accession number SRA018951. All simulated datasets are freely available from Zenodo (Weiner & Bansal, 2023b). The software used for our analysis is freely available open-source from https://github.com/samsonweiner/DICE and https://compbio.engr.uconn.edu/software/dice/.
